# Ageing and amyloidosis underlie the molecular and pathological alterations of tau in a mouse model of familial Alzheimer’s disease

**DOI:** 10.1038/s41598-019-52357-5

**Published:** 2019-10-31

**Authors:** Athanasios Metaxas, Camilla Thygesen, Stefan J. Kempf, Marco Anzalone, Ramanan Vaitheeswaran, Sussanne Petersen, Anne M. Landau, Hélène Audrain, Jessica L. Teeling, Sultan Darvesh, David J. Brooks, Martin R. Larsen, Bente Finsen

**Affiliations:** 1 Institute of Molecular Medicine, University of Southern Denmark, Odense C, Denmark; 2 Department of Biochemistry and Molecular Biology, University of Southern Denmark, Odense M, Denmark; 3 Department of Nuclear Medicine and PET-Centre, Aarhus University, Aarhus, Denmark; 4 Translational Neuropsychiatry Unit, Aarhus University, Aarhus, Denmark; 5 Biological Sciences, University of Southampton, Southampton, United Kingdom; 6 Department of Medical Neuroscience, Dalhousie University, Halifax, NS Canada; 7 Department of Medicine (Neurology and Geriatric Medicine), Dalhousie University, Halifax, NS Canada; 8 Division of Neuroscience, Faculty of Medical Science, University of Newcastle upon Tyne, Newcastle upon Tyne, UK

**Keywords:** Alzheimer's disease, Alzheimer's disease

## Abstract

Despite compelling evidence that the accumulation of amyloid-beta (Aβ) promotes neocortical MAPT (tau) aggregation in familial and idiopathic Alzheimer’s disease (AD), murine models of cerebral amyloidosis are not considered to develop tau-associated pathology. In the present study, we show that tau can accumulate spontaneously in aged transgenic *APP*_*swe*_/*PS1*_*ΔE9*_ mice. Tau pathology is abundant around Aβ deposits, and further characterized by accumulation of Gallyas and thioflavin-S-positive inclusions, which were detected in the *APP*_*swe*_/*PS1*_*ΔE9*_ brain at 18 months of age. Age-dependent increases in argyrophilia correlated positively with binding levels of the paired helical filament (PHF) tracer [^18^F]Flortaucipir, in all brain areas examined. Sarkosyl-insoluble PHFs were visualized by electron microscopy. Quantitative proteomics identified sequences of hyperphosphorylated and three-repeat tau in transgenic mice, along with signs of RNA missplicing, ribosomal dysregulation and disturbed energy metabolism. Tissue from the frontal gyrus of human subjects was used to validate these findings, revealing primarily quantitative differences between the tau pathology observed in AD patient vs. transgenic mouse tissue. As physiological levels of endogenous, ‘wild-type’ tau aggregate secondarily to Aβ in *APP*_*swe*_/*PS1*_*ΔE9*_ mice, this study suggests that amyloidosis is both necessary and sufficient to drive tauopathy in experimental models of familial AD.

## Introduction

Genetically-inherited and sporadic forms of Alzheimer’s disease (AD) are characterized by a common set of hallmark brain lesions, which include the aggregation of amyloid-β (Aβ) peptide into fibrillar plaques, neuroinflammation, aggregation of hyperphosphorylated MAPT (tau) protein into neurofibrillary tangles (NFTs), and neurodegeneration. Transgenic mouse models that reproduce the amyloid aggregation and glial activation due to Aβ overexpression have been generated based on mutations in the amyloid precursor protein (*APP*) and presenilin 1 (*PSEN1*) and 2 (*PSEN2*) genes, which are known to cause familial AD^1^. Despite playing important roles in evaluating APP processing, Aβ toxicity and amyloid-targeting therapeutic strategies, transgenic mice are not being regarded as models that can replicate the full spectrum of AD histopathology^2^. In particular, while the overexpression of mutant *APP* and *APP*/*PSEN1* yields amyloidosis^3^, neuroinflammation^4^ and degeneration of monoaminergic^5^ and cholinergic^6^ neurons, it is generally not considered sufficient to cause aggregation of endogenous murine tau into neurofibrillary structures^7^.

To address the role of tau hyperphosphorylation and NFT formation in AD pathogenesis, human *MAPT* (*TAU*) has been introduced into the mouse genome, either mutated or non-mutated, on a *tau*-knockout background^8,9^. *TAU* expressing mice demonstrate progressive neurofibrillary pathology, albeit in the absence of cerebral amyloidosis which is required for a neuropathological diagnosis of AD. Moreover, mutations in *TAU* have been linked to non-AD tauopathies, most commonly frontotemporal lobar degeneration [FTLD^10^], a condition with neuropathological hallmarks distinct from AD. Thus, murine models of amyloidosis and combined amyloidosis-tauopathy models have been widely criticized for their translational relevance to human AD. It has been argued that virtually all existing murine models would be considered as ‘not’ AD^11^ according to the ABC scoring system of neuropathology^12^. The inability of amyloidosis mice to develop neurofibrillary tau lesions is thought to contribute to the poor translation of preclinical research into clinical benefits^13^, and has raised concern about the amyloidocentric model of AD pathogenesis^14^.

Several factors have been proposed to account for the lack of tau-associated pathology in amyloidosis models. It has been suggested that the development of tauopathy in AD requires an imbalance in the expression of tau protein isoforms containing three (3R) and four (4R) microtubule-binding repeat domains^15,16^. By predominantly expressing 4R tau in the brain^17^, adult mice might be less susceptible to tau accumulation than species expressing both 3R and 4R isoforms, such as humans^18^ and rats^19^. However, murine^20^, rat^21^ and human^22^ tau have been shown to readily fibrillize *in vitro* upon treatment with polyanionic factors, indicating that tau’s propensity for aggregation is neither isoform, nor species-dependent. In addition, hallmark post-translational modifications (PTMs) that are associated with the accumulation of fibrillar tau in AD, such as phosphorylation^23^, have been detected in the brain of transgenic mice^24^, including *APP*_swe_/*PS1*_ΔE9_ mice (Supplementary Table S1). A third reason that is often cited for the absence of tau pathology in amyloidosis models is that the murine lifespan may be too short for the complete sequence of neurofibrillary pathology to unfold in transgenic mice^25^. Although age scaling studies suggest otherwise^26^, the ageing factor has been neglected in the design of preclinical studies.

The present study was based on evidence that murine tau aggregates into paired helical filaments (PHFs) *in vitro*, becoming hyperphosphorylated during the course of amyloidosis in the transgenic mouse brain. We reasoned that murine models of familial AD may spontaneously develop neurofibrillary pathology, provided they are sufficiently aged. Using techniques that are complimentary in their ability to detect fibrillar tau, we report that cortical tau-associated pathology develops secondarily to amyloidosis in *APP*_swe_/*PS1*_ΔE9_ mice. The observed pathology is characterised by aggregation of hyperphosphorylated tau and participation of both 3R and 4R isoforms. These results show that it is possible to model the relationship between aberrant APP processing and tau pathology in a translationally-relevant manner.

## Results

### Neurofibrillary pathology in aged APP_swe_/PS1_ΔE9_ mice

Fresh-frozen brain sections from *APP*_swe_/*PS1*_ΔE9_ transgenic (TG) mice and their wild-type (WT) counterparts were processed along with human brain sections for the detection of neurofibrillary alterations with the Gallyas silver stain. Co-staining for amyloid and Gallyas was used to probe the relationship between amyloidosis and tau-associated pathology.

**Figure 1:**
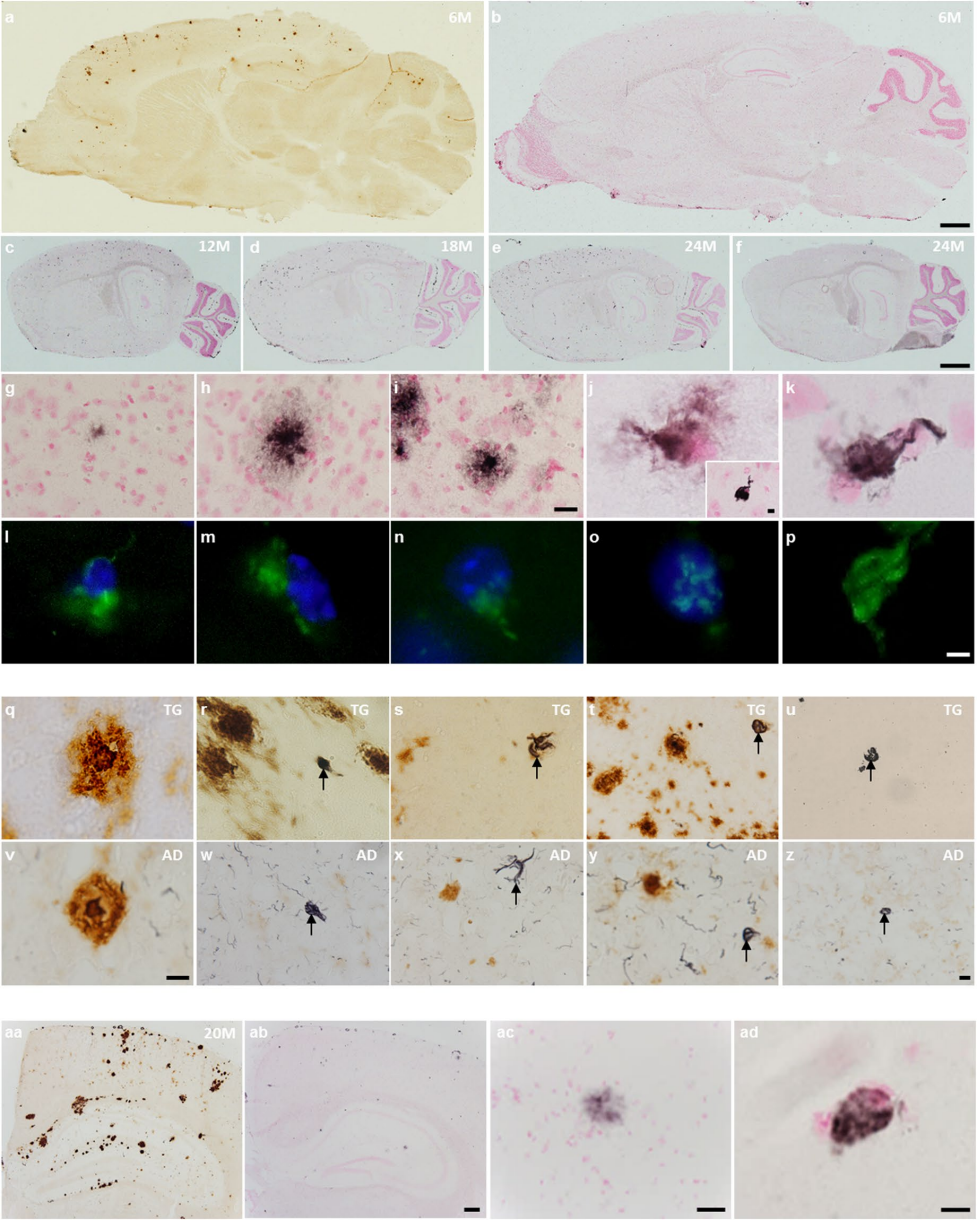
Neurofibrillary alterations in amyloidosis mice. a&b Sagittal brain sections of 6-month-old *APP*_swe_/*PS1*_ΔE9_ mice, processed for 6E10 immunohistochemistry (**a**) and the Gallyas silver stain (**b**). Silver-labelled sections were counterstained with nuclear fast red. β-amyloidosis dominates over argyrophilic pathology in the neocortex of 6-month-old *APP*_swe_/*PS1*_ΔE9_ mice. **(c**–**f)** Progressive increase in Gallyas-positive signal in 12 (**c**), 18 (**d**), and 24-month-old transgenic (TG) mice (**e**). Wild-type animals showed no silver deposition up to 24 months of age (**f**). **(g**–**p)** All photomicrographs are from the neocortex of *APP*_swe_/*PS1*_ΔE9_ mice_._ Argyrophilic signal was scarce in 6-month-old TG animals (**g**). Gallyas-positive structures in 18 (**h**) and 24-month-old animals (**i**), likely of neuritic nature. Gallyas silver (**j**,**k**) and thioflavin-S stainings (**l**–**p**), showing perinuclear and intranuclear signal in 18- and 24-month-old TG mice. The insert in j shows compact Gallyas staining in the absence of nuclear fast red. Note potential fragmented nuclei in (**m**) and (**n**), intranuclear signal in (**o**), and absence of DAPI signal in (**p**). **(q**–**z)** Gallyas/6E10 double-labelled sections from a 24-month-old TG mouse (**q**–**u**) and an AD patient (**v-z)**, showing dense-core plaques (**q**,**v**), teardrop-shaped structures (**r**,**w**, arrows), tuft-shaped filaments (**s,x**, arrows), and globose structures in close proximity (**t**,**y**) and over 200 µm afar from Aβ deposits (**u**,**z**). **(aa–ad)** 6E10/Gallyas- (aa) and Gallyas-labelled (ab-ad) sections of 20-month-old Tg2576 mice. Scale bar is 2 mm for a&b, 1 mm for c-f, 10 µm for g-i, 5 µm for j-p, 10 µm for q & v, 20 µm for r-u & w-z, 200 µm for aa&ab, 20 µm for ac, and 5 µm for ad.

Aβ deposition was the predominant lesion in the 6-month-old *APP*_swe_*/PS1*_ΔE9_ brain (Fig. 1a,b), with age-dependent increases in argyrophilic density observed exclusively in TG mice (Fig. 1c-f). Only mild and diffuse silver staining was observed in the neocortex of 6-month-old animals (Fig. 1g). Densely-labelled, plaque-like structures, surrounded by a halo of argyrophilic staining, constituted the majority of Gallyas-positive signal in the neuropil of the neocortex and hippocampus at 12–24 months of age (Fig. 1h,i). In addition, diffuse and compact argyrophilic staining was observed surrounding red-stained nuclei in the neocortex of 18 and 24-month-old *APP*_swe_*/PS1*_ΔE9_ mice (Fig. 1j,k). The perinuclear structures were positive for thioflavin-S (Fig. 1l–n). Thioflavin-S colocalized with nuclear DAPI (Fig. 1o) and was further detected in cell-sized structures lacking a stainable nucleus (Fig. 1p). There were no apparent differences in morphology between the argyrophilic structures in brain tissue from 24-month-old TG mice (Fig. 1q–u) and AD-confirmed patient material (Fig. 1v–z), although silver-stained neuropil threads were detected exclusively in AD tissue (Fig. 1q–z). Coronal brain sections of 20-month-old Tg2576 mice, harbouring the Swedish double mutations, were used to examine 6E10- and Gallyas-positive pathology in a second mouse model of amyloidosis (Fig. 1aa–ad). Amorphous argyrophilic signal (ac) and perinuclear lesions (ad) were detected in the Tg2576 mouse brain, albeit at lower levels compared to 18-month-old *APP*_swe_/*PS1*_ΔE9_ mice.

**Figure 2:**
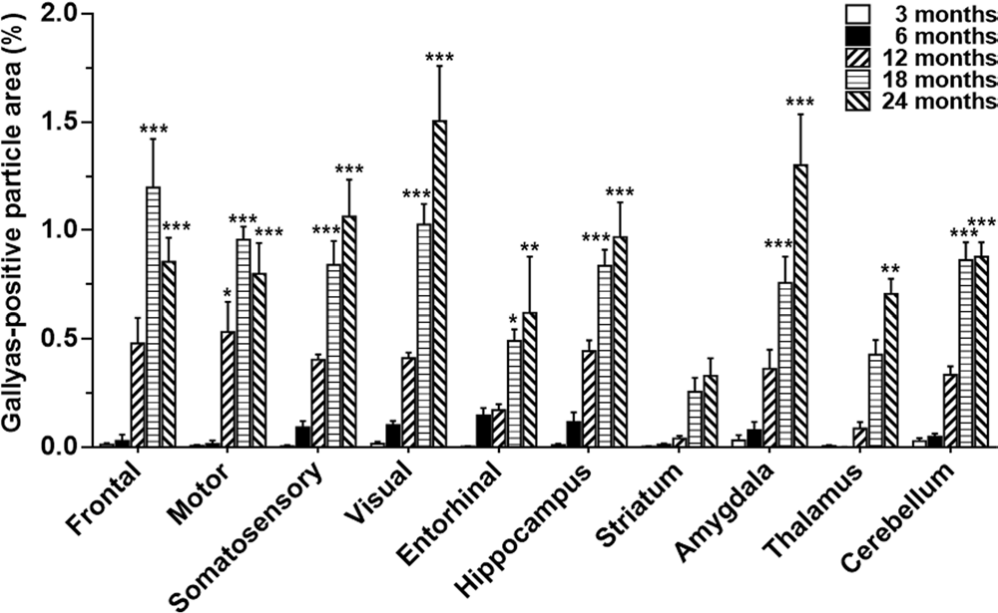
Quantification of Gallyas-positive signal in *APP*_swe_/*PS1*_ΔE9_ mice. Regions of interest were manually drawn by reference to the mouse brain atlas of Paxinos and Franklin^76^. Gallyas-positive particles were measured with the particle analysis plugin in ImageJ, after thresholding ROIs on a black and white image display mode, by using default software settings. Data are presented as the mean area fraction occupied by Gallyas-positive particles ± standard error of the mean (SEM), in brain regions of 5–6 animals/group. Asterisks denote increased argyrophilia compared to 3-month-old *APP*_swe_/*PS1*_ΔE9_ mice (*P < 0.05, **P < 0.01, ***P < 0.001, Bonferroni *post-hoc* tests). When all brain regions were analysed together, increased silver deposition was detected in 12 vs. 3 and 6-month-old *APP*_swe_*/PS1*_ΔE9_ mice (*P* < 0.001), with additional accumulation occurring in 18- vs. 12- (*P* < 0.001), and 24- vs. 18-month-old TG animals (*P* < 0.05, Bonferroni *post hoc* tests). Two-way ANOVA confirmed significant main effects of age [F_(4,245)_ = 169.9, *P* < 0.001] and brain region [F_(9,245)_ = 11.4, *P* < 0.001], as well as significant age × region interaction effects on the fraction of brain tissue bearing Gallyas-positive signal [F_(36,245)_ = 3.2, *P* < 0.001].

The fraction of brain tissue occupied by Gallyas-positive staining in *APP*_swe_/*PS1*_ΔE9_ mice is shown in Fig. 2. Approximately 4-fold higher levels of argyrophilic staining were measured in the positive control AD section (3.9%) compared to the 24-month-old *APP*_swe_/*PS1*_ΔE9_ neocortex (0.96%). Vascular and meningeal lesions were present in 18 and 24-month-old animals (Supplementary Fig. S1).

### Age-dependent increase in [^18^F]Flortaucipir binding levels in *APP*_swe_/*PS1*_ΔE9_ mice

**Figure 3:**
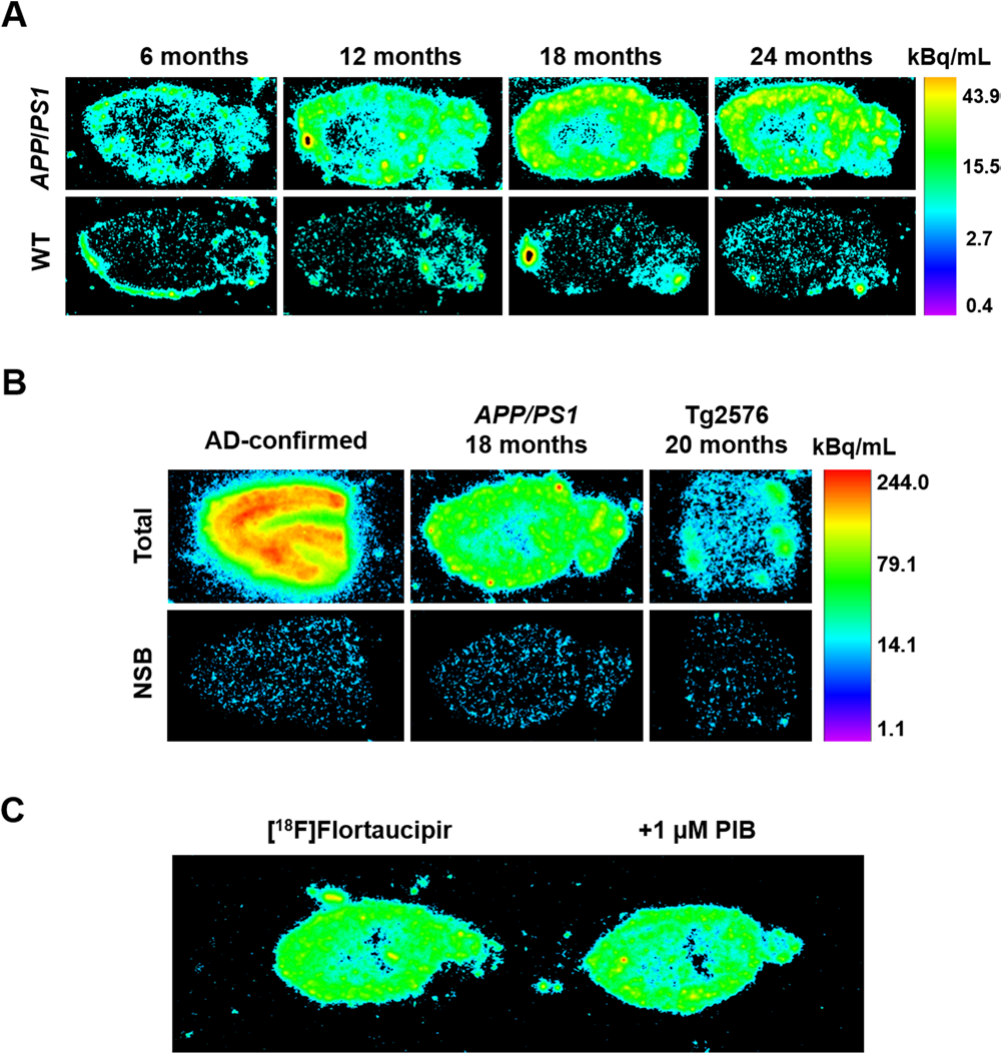
Representative autoradiograms of [^18^F]Flortaucipir binding sites. (**A**) Sagittal brain sections of transgenic (top panel) and wild-type mice (WT, lower panel), taken at the level of the entorhinal cortex [lateral 2.88 ± 0.12 mm of the Paxinos and Franklin mouse atlas^76^]. Images were analysed on a black & white display mode, and presented as a pseudocolor interpretation of black & white pixel intensity, calibrated in kBq/mL of [^18^F]Flortaucipir solution. Age-dependent increases in binding levels were observed exclusively in *APP*_swe_/*PS1*_ΔE9_ mice. **(B)** [^18^F]Flortaucipir binding in sections from the middle frontal gyrus of an AD-confirmed patient, 18-month-old *APP*_swe_/*PS1*_ΔE9_ mice and 20-month-old Tg2576 animals, showing the magnitude of tau pathology in patient vs. transgenic mouse tissue. Non-specific binding (NSB) was assessed in the presence of 50 µM ‘cold’ flortaucipir. **(C)** Binding was not blocked by co-incubating sections with [^18^F]Flortaucipir and 1 µM of the amyloid-targeting agent Pittsburgh Compound B (PIB).

The PHF ligand [^18^F]Flortaucipir ([^18^F]AV-1451, [^18^F]T807) was used to quantify tau pathology in *APP*_swe_/*PS1*_ΔE9_ TG mice by autoradiography (Table 1). Increased binding was observed in the neocortex, hippocampus, amygdala and the cerebellum of 12-month-old *APP*_swe_/*PS1*_ΔE9_ mice, compared to age-matched WT animals, and vs. 3 and 6-month-old TG mice (*P* < 0.001 for all regions; Bonferroni *post-hoc* tests). [^18^F]Flortaucipir binding was further elevated in the visual (*P* < 0.001), somatosensory (*P* < 0.001), motor cortex (*P* < 0.001), and the amygdala (*P* < 0.05) of 18 vs. 12-month-old *APP*_swe_/*PS1*_ΔE9_ TG mice. Increased binding over age-matched control mice was first observed in the thalamus of TG animals at 18 months of age (*P* < 0.001). In 24-month-old *APP*_swe_/*PS1*_ΔE9_ mice, [^18^F]Flortaucipir signal had increased in all brain regions examined compared to age-matched controls. Three-way ANOVA confirmed genotype- [F_(1,476)_ = 2603.1, *P* < 0.001], age- [F_(4,476)_ = 457.3, *P* < 0.001] and brain region-specific increases in the binding levels of [^18^F]Flortaucipir [F_(9,476)_ = 42.9, *P* < 0.001], as well as significant age × genotype × region interaction effects [F_(36,476)_ = 5.5, *P* < 0.001]. Representative autoradiograms of [^18^F]Flortaucipir binding sites are shown in Fig. 3(A–C). Binding was abolished in the presence of 50 µM unlabelled Flortaucipir (Fig. 3B), but was not blocked by 1 µM of the beta sheeted amyloid-marker Pittsburgh compound B (PIB; Fig. 3C). [^18^F]Flortaucipir binding levels in 3 consecutive sections of the AD-confirmed neocortex were 4-fold higher compared to the neocortex of 24-month-old *APP*_swe_/*PS1*_ΔE9_ mice (191.3 ± 7.1 kBq/mL vs. 53.6 ± 6.1 kBq/mL).

**Table 1:** Autoradiography of [^18^F]Flortaucipir binding sites in *APP*_swe_/*PS1*_ΔE9_ mice.

Brain region	3 months		6 months		12 months		18 months		24 months		Correlation with Gallyas-positive fraction
*Cortical*	WT	APP/PS1	WT	APP/PS1	WT	APP/PS1	WT	APP/PS1	WT	APP/PS1	Pearson r (Significance)
Frontal	2.2 ± 0.7	3.6 ± 1.3	2.3 ± 0.9	10.0 ± 1.6	4.0 ± 1.0	46.7 ± 2.7***	4.7 ± 1.1	53.2 ± 3.0	4.2 ± 1.0	55.2 ± 5.7	0.74 (*P* < 0.001)
Motor	2.5 ± 0.5	2.8 ± 0.8	2.5 ± 0.7	7.3 ± 1.3	3.2 ± 0.7	31.0 ± 1.3***	3.2 ± 0.8	45.1 ± 2.0	4.3 ± 1.1	55.7 ± 2.9	0.90 (*P* < 0.001)
Somatosensory	5.2 ± 1.3	5.0 ± 2.0	4.0 ± 0.9	11.6 ± 1.9	6.7 ± 4.5	34.9 ± 2.6***	6.5 ± 1.8	50.3 ± 1.5	5.8 ± 1.6	57.8 ± 2.7	0.93 (*P* < 0.001)
Visual	6.8 ± 2.4	4.7 ± 1.4	2.4 ± 1.1	11.8 ± 2.5	8.2 ± 1.8	35.5 ± 3.2***	6.3 ± 1.9	52.9 ± 2.8	5.0 ± 1.3	56.4 ± 2.9	0.92 (*P* < 0.001)
Entorhinal	3.2 ± 1.0	2.9 ± 1.0	3.3 ± 0.8	8.8 ± 1.1	6.7 ± 1.5	29.8 ± 1.9***	4.1 ± 2.6	40.6 ± 3.8	6.3 ± 1.7	42.8 ± 4.0	0.84 (*P* < 0.001)
***Subcortical***											
Hippocampus	3.1 ± 1.3	2.8 ± 0.8	3.0 ± 0.7	7.5 ± 1.3	4.3 ± 1.3	31.3 ± 2.4***	4.3 ± 1.2	40.9 ± 2.5	5.6 ± 1.5	49.1 ± 3.1	0.86 (*P* < 0.001)
Striatum	2.6 ± 1.4	3.1 ± 1.0	3.1 ± 0.7	4.8 ± 0.9	4.4 ± 1.1	10.9 ± 2.4	5.1 ± 1.5	17.2 ± 1.2	5.4 ± 1.5	19.7 ± 2.8**	0.64 (*P* < 0.001)
Amygdala	1.8 ± 1.5	2.9 ± 0.9	3.1 ± 0.7	7.4 ± 1.0	4.2 ± 1.1	27.8 ± 2.9***	5.2 ± 1.5	40.0 ± 2.5	5.2 ± 1.1	47.7 ± 4.8	0.79 (*P* < 0.001)
Thalamus	2.6 ± 0.6	2.2 ± 0.6	3.3 ± 1.0	3.5 ± 0.5	3.6 ± 0.9	11.2 ± 3.2	3.9 ± 1.3	17.5 ± 2.0***	4.1 ± 1.0	27.2 ± 4.5	0.87 (*P* < 0.001)
Cerebellum	4.2 ± 0.8	3.1 ± 1.1	4.1 ± 1.2	10.3 ± 1.1	3.3 ± 1.0	23.0 ± 3.1***	4.5 ± 1.5	31.2 ± 3.6	5.8 ± 1.6	32.4 ± 3.7	0.83 (*P* < 0.001)
Mean binding levels (all brain regions)	3.4 ± 1.1	3.3 ± 1.1	3.1 ± 0.9	8.3 ± 1.3	4.9 ± 1.5	28.2 ± 2.6	4.8 ± 1.5	38.9 ± 2.5	5.2 ± 1.3	44.4 ± 3.7	0.92 (*P* < 0.001)

Fresh-frozen brain sections from *APP*_swe_/*PS1*_ΔE9_ and age-matched wild-type (WT) animals were incubated with 38.4 ± 9.6 MBq [^18^F]Flortaucipir for a period of 60 min (specific activity: 145 ± 68 GBq/µmol). Autoradiography data are presented as the mean specific binding of [^18^F]Flortaucipir (kBq/mL) ± standard error of the mean in brain regions of 5–6 animals/group. By 24 months of age, [^18^F]Flortaucipir binding in *APP*_swe_/*PS1*_ΔE9_ mice had increased across all brain areas examined compared to age-matched WT animals. The age-dependent increase in [^18^F]Flortaucipir binding levels was positively correlated with the progressive increase in Gallyas-positive argyrophilic signal, in all TG brain areas examined. **P < 0.01, ***P < 0.001 vs. age-matched littermate control mice, Bonferroni *post-hoc* tests. Symbols of significant differences between groups of 24 & 18 vs. 3, 6 and 12-month-old-mice were omitted from the table for clarity of presentation.

Within each brain area analysed, there was a positive correlation between the age-dependent increase in the binding levels of [^18^F]Flortaucipir and the progressive increases in the density of Gallyas-positive lesions (Pearson r for all brain regions: 0.92, *P* < 0.001; Table 1).

### Unaltered Mapt expression in ageing APP_swe_/PS1_ΔE9_ mice

Relative expression of total *Mapt* mRNA was determined by RT-qPCR (Supplementary Fig. S2). There were no age [F_(4,50)_ = 0.29, *P* > 0.05], genotype [F_(1,50)_ = 0.93, *P* > 0.05], or age × genotype interaction effects on the expression levels of *Mapt* [F_(4,50)_ = 1.21, *P* > 0.05].

### Isolation and transmission electron microscopy (TEM) of sarkosyl-insoluble tau

The general methods of Sahara *et al*.^27^ and Greenberg and Davies^28^ were evaluated for the extraction of PHFs from the 24-month-old TG brain (Supplementary Fig. S3). Although longer filaments were isolated by the procedure of Sahara *et al*., the Greenberg and Davies method was chosen for the isolation of sarkosyl-insoluble tau from 3 and 24-month-old mice, based on immunoblotting experiments, solubility considerations, and to allow for comparisons with literature data^29^. Soluble and insoluble tau levels were measured in mouse brain homogenates by using the mouse Total Tau Meso Scale kit (Meso Scale Diagnostics LLC). TEM was used to visualize filaments in the sarkosyl-insoluble extracts from the mouse and AD patient brains by negative staining.

**Figure 4:**
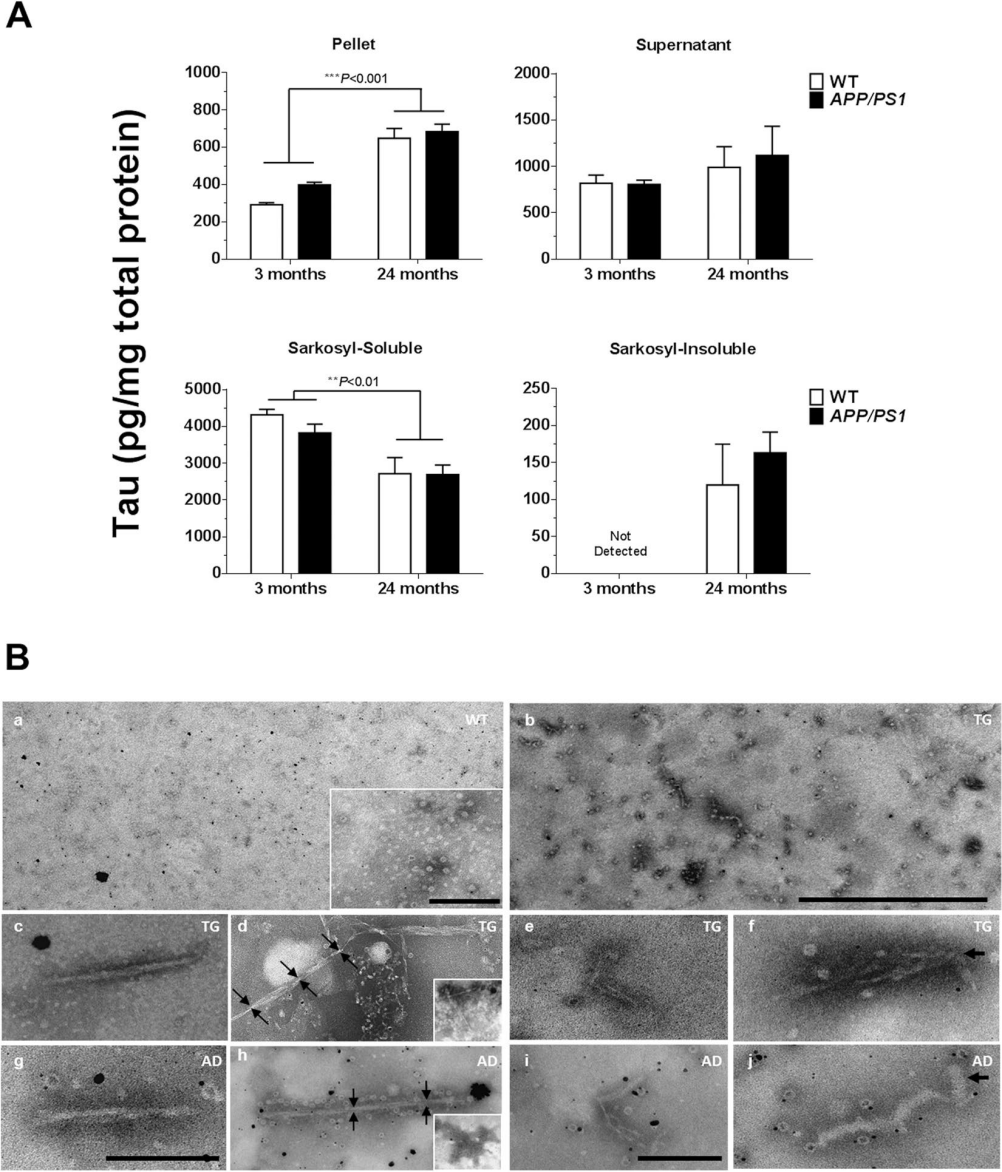
Isolation and electron microscopy of sarkosyl-insoluble tau. (**A**) Levels of soluble and insoluble tau were determined with the mouse Total Tau Meso Scale kit. Tau levels increased with age in the pellet obtained by centrifuging brain homogenates at 27,000 × g. The resulting supernatant was treated with 1% sarkosyl and centrifuged at 200,000 × g. The solubility of tau in sarkosyl decreased with age, irrespective of genotype. **(B)** Overview of negatively-stained material in sarkosyl-insoluble lysates from (a) aged wild-type (WT) and (b) aged *APP*_swe_/*PS1*_ΔE9_ transgenic (TG) mice. Fibrils of ~20 nm in width, appearing as straight filaments (c) or as two intertwined fibrils (g), each with a diameter of ~10 nm. PHFs with axial periodicities of ~80 nm were present in *APP*_swe_/*PS1*_ΔE9_ mice (d; arrows), and more frequently observed in AD patient material (h; arrows). The inserts show ‘stacked’ PHFs, which were denser in the AD preparation. Structures identified in the detergent-insoluble fractions of the mouse and human brain included bent fibrils of ~7 nm in width (e,i), and rod-shaped particles (arrows e & f; insert a). Scale bars: 1 µm (a, b); 200 nm (c,d,g,h); 100 nm (e,f,i,j; insert a).

Tau protein levels increased with age in the pellet obtained by centrifuging WT and *APP*_swe_/*PS1*_ΔE9_ homogenates at 27,000 × g [Fig. 4A; age effect: F_(1,18)_ = 50.0, *P* < 0.001; genotype effect: F_(1,18)_ = 2.4, *P* > 0.05]. Levels of tau in the supernatant fraction were not different between 3 and 24-month old, WT and *APP*_swe_/*PS1*_ΔE9_ TG mice [age: F_(1,16)_ = 0.6, *P* > 0.05; genotype: F_(1,16)_ = 0.0, *P* > 0.05]. Treatment of the supernatant with 1% sarkosyl for 2 h at 37 °C increased the concentration of tau in the detergent-soluble fraction by > 3-fold. Sarkosyl-soluble tau levels were lower in the 24 vs. 3-month-old mouse brain [F_(1,16)_ = 12.5, *P* < 0.01], irrespective of genotype [F_(1,16)_ = 0.5, *P* > 0.05]. Sarkosyl-insoluble tau was not detected in 3-month-old animals, and its levels were not different between 24-month-old *APP*_swe_/*PS1*_ΔE9_ and WT mice [t_(8)_ = 0.7, *P* > 0.05; independent two-tailed Student’s t-test].

Negative stains of sarkosyl-insoluble lysates from 24-month-old mice and AD patient brain are shown in Fig. 4B. No filaments were observed in 24-month-old WT mice (a). Fibrils of mean length 104.9 ± 8.3 nm and width 10.1 ± 0.5 nm were isolated from aged TG mice (b). Wider fibrils (~20 nm), with or without a pronounced twist, were readily detected in both TG (c) and AD patient material (g). Longer filaments (271.7 ± 11.3 nm), with axial periodicities of 78.7 ± 9.8 nm, constituted ~8% and ~34% of the fibril population analysed in the *APP*_swe_/*PS1*_ΔE9_ (d) and AD brains (h), respectively. Clusters of long filaments, which were denser in AD patient material, were present in the insoluble preparation from *APP*_swe_/*PS1*_ΔE9_ mice (d & h, inserts). There were no between-species differences in the dimensions of the isolated filaments [short filaments, length: t_(82)_ = 0.1, *P* > 0.05, width: t_(82)_ = 1.2, *P* > 0.05; long filaments, length: t_(16)_ = 0.3, *P* > 0.05, width: t_(16)_ = 0.8, *P* > 0.05; independent two-tailed t-tests]. In addition to straight and helical filaments, thin/bent fibrils (e & i) and rod-shaped particles (f & j) were observed in both 24-month-old TG mice (e & f) and AD brains (i & j). Rod-shaped, granular particles were observed in two of three independent experiments using lysates from aged WT mice.

### Proteomics of sarkosyl-insoluble tau

The sarkosyl-insoluble fractions extracted from 3 and 24-month-old mouse brain, AD and non-AD material, were digested with trypsin & Lys-C. The peptides were labelled with Tandem Mass Tags (TMT), pooled 1:1, fractionated, and analysed by nanoLiquid Chromatography-Electrospray Ionization Mass Spectrometry (LC-ESI MS/MS). A list of tau-associated proteins quantified in the sarkosyl-insoluble proteome is shown in Table 2. Lists of all identified proteins and between-group abundance ratios are provided as a Supplementary Dataset.

**Table 2:** Proteomics of sarkosyl-insoluble tau.

UniProt	Protein (Sequence)	Involvement	TG vs. WT	TG vs. WT	TG		WT		AD vs. non-AD
Accession Number			3 M	24 M	24 vs. 3 M		24 vs. 3 M		
B1AQW2	Microtubule-associated protein	Tau	0.86	1.00	2.06		1.77		37.15
	(KVAVVRTPPKSPSASKS)	pT231	0.87	26.55	23.81		0.78		0.94
	(KSPVVSGDTSPRH)	pS396, pS400, pS404	1.46	2.02	1.00		0.67		1.16
P10637-3	Microtubule-associated protein tau	Tau Isoform-B	0.61	3.21	4.52		0.86		Fetal form present
Multiple	Small nuclear ribonucleoproteins (SnRPN)	Core spliceosomal components	Age- and genotype-specific regulation (Supplementary Dataset)						
Multiple	Heterogeneous nuclear ribonucleoproteins	Exon 10 splicing regulation	Multiple proteins regulated (Supplementary Dataset)						
Q8BL97	Serine/arginine-rich splicing factor 7	Exon 10 exclusion	0.82	1.05	1.11	0.87		0.69	
Q9Z0H4	CUGBP Elav-like family member 2	Exon 10 exclusion	0.40	1.85	1.94	0.42		1.49	
P62996	Transformer-2 protein homolog beta	Exon 10 inclusion	0.85	1.13	0.74	0.56		Not identified	
Multiple	Tubulin	Tau binding partner	Alpha & beta chains regulated (Supplementary Dataset)						
P60710	Actin, cytoplasmic 1	Tau binding partner	0.80	0.53	1.43	2.16		0.71	
P08551, P08553, P19246	Neurofilament	Tau binding partner	Light, medium & heavy polypeptides regulated (Supplementary Dataset)						
Multiple	Ribosomal proteins 60 S, 40 S	Tau binding partner	Age- and genotype-specific changes, particularly in the acidic proteins of the 60 S subunit (Supplementary Dataset)						
O08788	Dynactin	Tau binding partner	1.01	1.34	0.79	0.59		Not identified	
P28738	Kinesin	Tau binding partner	0.92	0.75	1.03	1.28		0.90	
P11499, Q80Y52, Q3UAD6	Heat shock protein 90	Tau binding partner	Isoforms alpha & beta identified (Supplementary Dataset)						
P48722, P17156, Q3U2G2, Q8K0U4	Heat shock protein 70	Tau binding partner	Members 2 & 4 common in mice & humans (Supplementary Dataset)						
P0DP26	Calmodulin-1	Tau binding partner	1.68	0.31	0.68	3.63		0.93	
Q3UY00	S100β	Tau binding partner	0.99	0.52	1.27	2.44		0.34	
O55042	α-Synuclein	Tau binding partner	0.81	0.70	1.29	1.49		0.79	
A8IP69, P68510, F6VW30, Q9CQV8, P63101	14-3-3 proteins	Tau binding partner	Isoform-specific changes (Supplementary Dataset)						
Q3TXU4	Apopoliprotein E	Tau binding partner/AD risk factor	0.65	3.58	7.51	1.37		Not identified	
O08539	Bin 1	Tau binding partner/AD risk factor	2.11	0.44	0.71	3.41		1.44	
Q549A5	Clusterin	Tau interacting partner AD risk factor	1.17	1.82	1.87	1.20		Not identified	
P11798, Q923T9, A0A0G2JGS4	Ca2+/calmodulin-dependent protein kinase II	Tau kinase	Multiple subunits identified (Supplementary Dataset)						
P63318	Protein kinase C, gamma type	Tau kinase	0.71	1.48	1.30	0.62		1.05	
P31324	Cyclic-AMP dependent protein kinase II	Tau kinase	0.57	0.91	1.61	1.01		0.84	
Q63810	Calcineurin subunit B type I	Tau phosphatase	1.36	0.34	0.96	3.84		Not identified	
Q76MZ3	Serine/threonine-protein phosphatase 2A	Tau phosphatase	1.25	0.58	0.87	1.88		0.73	

Tau-associated proteins quantified in the detergent-insoluble fractions of the mouse and human brain. The presented proteins have been selected for their documented roles in the regulation and binding of tau. Phosphorylation sites are reported according to the human isoform of tau with 441 amino acids.

There were 583 proteins identified in the sarkosyl-insoluble mouse proteome, of which 456 were also present in the human samples. Isoforms of tau with three (3R) and four (4R) microtubule-binding repeats were extracted from both human and the murine brain. In mice, all tau isoforms collapsed under the term microtubule associated protein (MAP; UniProt accession number: B1AQW2). Mouse MAP was regulated by age, rather than genotype. The protein was enriched 2.1-fold in 24 vs. 3-month-old TG mice, and 1.8-fold in 24 vs. 3-month-old WT mice. Individual isoforms of tau were differentially regulated between *APP*_swe_/*PS1*_ΔE9_ and WT mice. Tau isoform-B (UniProt accession number: P10637-3), containing the motif ^205^KVQIVYKPVDLSKV^218^, is a 3R isoform with an extended C-terminal domain, which was increased 3.2-fold in 24-month-old TG vs. WT mice, and 4.5-fold in 24 vs. 3-month-old TG animals. Human MAP (UniProt accession number: A0A0G2JMX7), containing tau isoforms P10637-2, -4, -6 & -8, was 37-fold enriched in the sarkosyl-insoluble fraction of AD compared to non-AD brain.

Although levels of unmodified tau were not different between age-matched WT and TG mice, the mouse MAP sequence ^174^KVAVVR**T**PPKSPSA**S**KS^190^, phosphorylated at Threonine (T) 180 and Serine (S) 188, was more than 20-fold enriched in 24-month-old *APP*_swe_/*PS1*_ΔE9_ mice compared to age-matched WT and 3-month-old TG mice. The hyperphosphorylated sequence was not regulated in 24 vs. 3-month-old WT animals. An orthologous sequence of the human MAP was phosphorylated at Threonine (T) 566 and Serine (S) 573 (^560^KVAVVR**T**PPKSPS**S**AKS^576^). The reported phosphorylation sites correspond to amino acids (aa) T231 and S238 of the human tau isoform with 441 aa. Indications of additional phosphorylation sites were obtained by searching modified peptides against tau isoform- and species-specific databases. Phosphorylated S396, S400 and S404 on the conserved sequence ^396^**S**PVV**S**GDT**S**PR^406^ of the human 441 aa isoform were identified in the sarkosyl-insoluble mouse proteome, and were > 2.0-fold enriched in 24-month-old TG vs. WT mice. In addition to phosphorylation, murine MAP was deamidated at Asparagine (N) 44, a site on the N-terminal domain of tau that is not conserved in humans (^34^AEEAGIGDTP**N**QEDQAAGHVTQAR^57^). Human MAP was deamidated at position N484, corresponding to N167 of the 441 aa tau isoform (^473^GAAPPGQKGQA**N**ATRIPAK^491^).

**Figure 5:**
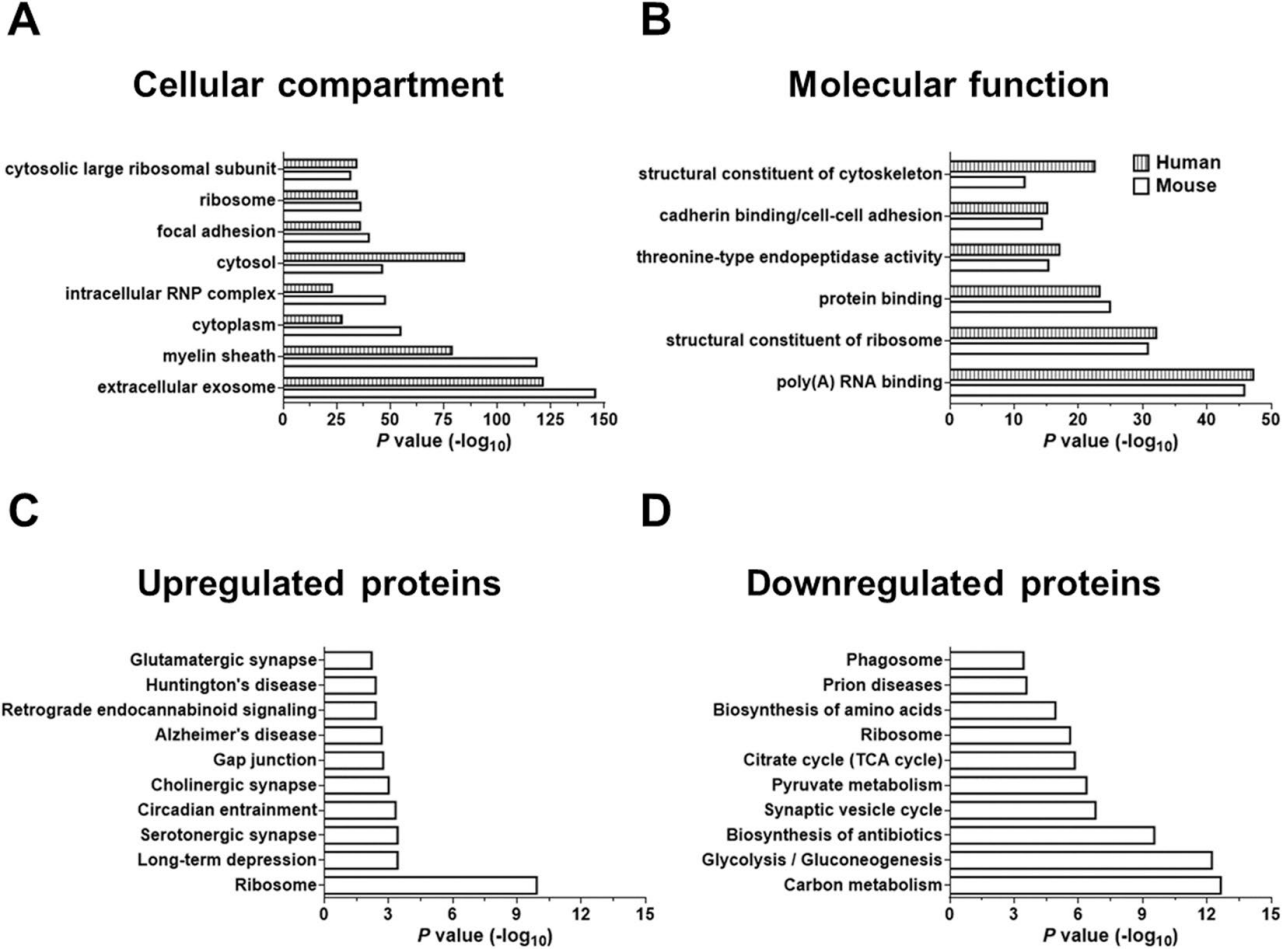
Gene Ontology (GO) enrichment analysis of the sarkosyl-insoluble proteome. (**A)** Enriched cellular components; **(B)** Enriched molecular functions; **(C)** Top 10 enriched pathways based on protein upregulation in 24-month-old TG vs. WT mice, according to the Kyoto Encyclopaedia of Genes and Genomes (KEGG); **(D)** Top 10 enriched KEGG pathways based on protein downregulation in 24-month-old TG vs. WT mice. Functional annotation clustering was generated by using DAVID software. Maximum enrichment probability (*P* value) was based on an EASE score threshold value of 0.05.

The database for annotation, visualization and integrated discovery (DAVID, v6.8) was used for gene ontology (GO) enrichment analysis of the sarkosyl-insoluble proteome^30,31^. RNA splicing, mRNA processing and translation were among the 10 most enriched biological processes associated with protein upregulation in 24-month-old *APP*_swe_/*PS1*_ΔE9_ vs. WT mice and AD vs. non-AD subjects. Ribonucleoprotein complexes, ribosomes, and exosomes were among the 10 most enriched cellular components in the insoluble extracts from the mouse and human brain (Fig. 5A). The top 10 molecular functions of the enriched proteins were associated with poly(A) RNA binding, as well as binding of molecules contributing to the structural integrity of ribosomes and the cytoskeleton (Fig. 5B). Pathway-based enrichment analysis of upregulated proteins in 24-month-old *APP*_swe_/*PS1*_ΔE9_ vs. WT mice involved GO terms such as Alzheimer’s and Huntington’s disease, long-term depression, cholinergic, serotonergic and glutamatergic synapse (Fig. 5C). Glycolysis/gluconeogenesis and the Krebs cycle were among the top 10 pathways for downregulated proteins (Fig. 5D).

### Validation of pS404, pT231 and 3R tau

**Figure 6:**
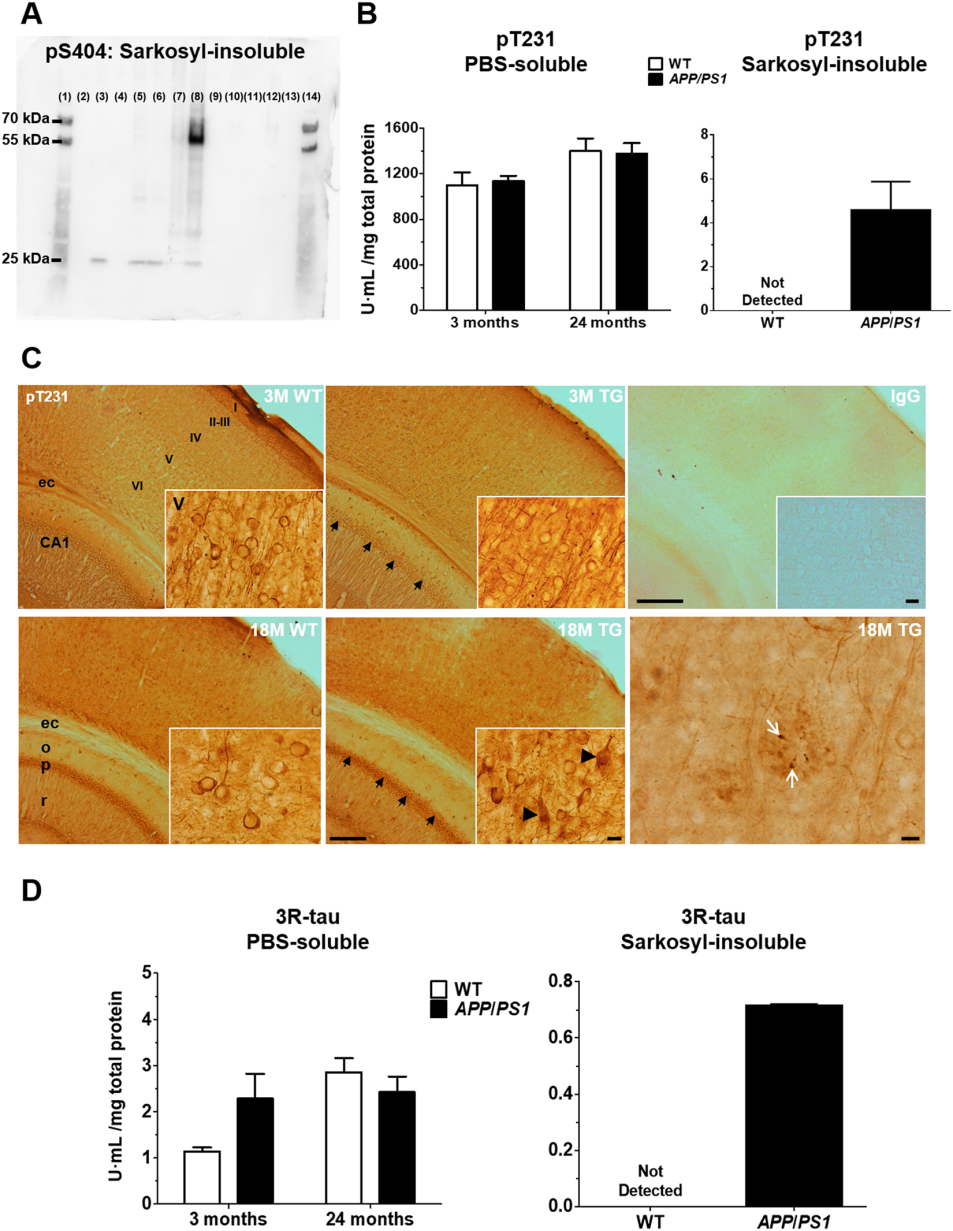
Validation of pS404, pT231 and 3R tau. (**A)** Immunoblotting of sarkosyl-insoluble tau with rabbit primary antibody against phospho-Ser404 (1:200; OAAF07796, Aviva Systems Biology). The entire membrane is shown. Hyperphosphorylation at the S404 residue was exclusively observed in 24-month-old *APP*_swe_*/PS1*_ΔE9_ mice (TG 24 M, lane 8). Lanes are labelled as follows: Marker: 1, 14; AD: 3; non-AD: 5; WT 24 months: 6; TG 24 months: 8; WT 3 months: 10; TG 3 months: 12; Empty: 2, 4, 7, 9, 11, 13. **(B)** ELISA of pT231 tau. Soluble pT231 tau was present in the neocortex of both WT and TG mice. Phosphorylation at T231 was only observed in the sarkosyl-insoluble fraction from 24-month-old *APP*_swe_*/PS1*_ΔE9_ mice. Results are expressed as arbitrary units (U), normalized to total protein concentration. **(C)** Immunohistochemistry of pT231 tau in coronal, 50 µm-thick brain sections from 3- and 18-month-old WT and TG mice. Black arrows point to CA1 pyramidal neurons, which were more strongly immunolabelled in 18 vs. 3-month-old animals, irrespective of genotype. Scale bar: 200 µm. The inserts show higher magnifications of layer V neurons in the temporal cortex, with black arrowheads pointing to tangle-like structures in aged *APP*_swe_*/PS1*_ΔE9_ mice. Note reduced dendritic staining in 18 vs. 3-month-old animals. Scale bar: 10 µm. White arrows point to puncta of pT231 immunoreactivity within a plaque-like structure, observed exclusively in the neocortex of TG mice. Scale bar: 20 µm. Abbreviations: CA1: cornu ammomis field 1; ec: external capsule; o- striatum oriens; p: striatum pyramidalis; r: striatum radiatum. **(D)** ELISA of 3R tau. Low levels of soluble 3R tau were present in the neocortex of both WT and TG animals. Sarkosyl-insoluble 3R tau was only observed in 24-month-old *APP*_swe_*/PS1*_ΔE9_ mice. Results are expressed as arbitrary units (U), normalized to total protein concentration.

Phosphorylation at the S404 residue of sarkosyl-insoluble tau was confirmed in 24-month-old *APP*_swe_/*PS1*_ΔE9_ mice by immunoblotting (Fig. 6A). New samples were generated to compare the levels of soluble and insoluble, pT231 and 3R tau in TG vs. WT mice. Levels of soluble pT231 increased with age in the neocortex [F_(1,20)_ = 8.4, *P* < 0.01], irrespective of genotype [F_(1,20)_ = 0.0, *P* > 0.05; Fig. 6B]. The effect was small, and individual Bonferroni comparisons showed no differences in pT231 tau concentration between 3 and 24-month-old mice (*P* > 0.05). Sarkosyl-insoluble pT231 tau was observed exclusively in 24-month-old TG animals (Fig. 6B). Immunohistochemistry of pT231 tau in free-floating sections from 3- and 18-month-old WT and TG mice is shown in Fig. 6C. Stronger immunoreactivity was observed in the cell soma of 18 vs. 3-month-old animals. Additionally, pT231 immunoreactive puncta were observed within plaque-like structures, exclusively in the neocortex of aged TG mice. For 3R tau (Fig. 6D), low levels of soluble protein were identified in the neocortex of both TG and WT mice by ELISA. There was no genotype effect on the concentration of soluble 3R tau [F_(1,20)_ = 0.9, *P* > 0.05]. Ageing increased 3R tau levels in the neocortex [F_(1,20)_ = 6.8, *P* < 0.05], but the effect was small, and Bonferroni *post-hoc* tests showed no differences in 3R tau concentration between 24 and 3-month-old animals (*P* > 0.05). In the sarkosyl-insoluble fraction, 3R tau was only observed in 24-month-old TG mice. RT-qPCR data of *Mapt* isoform-B mRNA and immunoblotting of PBS-soluble 3R tau are shown in Supplementary Fig. S4.

## Discussion

Tau protein hyperphosphorylation has been observed in various regions of the *APP*_swe_/*PS1*_*dE9*_ brain. The present study confirms and extends this literature, by showing that hyperphosphorylated tau converts into neurofibrillary structures in aged transgenic *APP*_swe_/*PS1*_*dE9*_ and Tg2576 mice. Neurofibrillary alterations in these mice were demonstrated by a set of tools that preferentially label insoluble tau in human AD, such as the Gallyas silver stain^32^ and [^18^F]Flortaucipir^33^. Straight and helical filaments were visualised by TEM of sarkosyl-insoluble preparations from the 24-month-old *APP*_*swe*_/*PS1*_*ΔE9*_ brain. As murine tau possesses a remarkably high number of potential serine/threonine (76) and tyrosine (4) phosphorylation sites, an unbiased proteomics approach was used for the identification and quantification of hyperphosphorylated tau epitopes. Of the five residues identified, pT231 and pS404 have been associated with early and late stages of tau aggregation, respectively^34,35^. The pathology observed in the present study occurred at physiological levels of endogenous tau, as there was no difference in total tau mRNA and protein between *APP*_*swe*_/*PS1*_*ΔE9*_ and WT mice. These results indicate that, in addition to amyloidosis^3^, neuroinflammation^4^ and neurodegeneration^5^, *APP*_*swe*_/*PS1*_*ΔE9*_ mice exhibit face validity for the neurofibrillary lesions of AD.

*Post-mortem*^36^ and *in vivo* imaging data^37^ reveal that the development of cortical tau pathology in AD patients is associated with, and may depend on, pre-existing β-amyloid plaque pathology. Aβ deposition has also been shown to precede neurofibrillary pathology in rat models of overexpression^19^ and inducible expression^38^ of mutant *APP*/*PS1*. Supporting this continuum of AD pathology, increased [^18^F]Flortaucipir binding levels and elevated argyrophilic signal succeeded the deposition of neocortical Aβ in *APP*_swe_/*PS1*_*dE9*_ mice. Gallyas- and thioflavin-S-positive inclusions, in particular, were observed by 18 months of age, when Aβ plaque load has been shown to plateau in the neocortex of *APP*_swe_/*PS1*_*dE9*_ mice^3^. These data suggest that the amyloidogenic processing of APP is sufficient to induce tau pathology in models of familial AD. The central role of amyloidosis is further supported by the fact that ageing alone did not induce neurofibrillary lesions in wild-type animals. In agreement with cross-sectional data showing age-dependent accumulation of tau in cognitively unimpaired subjects^39–42^, tau solubility was decreased in 24 vs. 3-month-old wild-type mice. However, a combination of fibrillar, insoluble and hyperphosphorylated tau was exclusively observed in *APP*_swe_/*PS1*_*dE9*_ mice. These data show that age-related tau accumulation can be distinguished from AD tau within the murine life-span, with cerebral amyloidosis driving the distinction in models of familial AD.

Phosphorylation is the most extensively studied tau PTM^43^. Evidence suggests that the phosphorylation pattern of specific tau epitopes is associated with distinct morphological stages of NFT formation. Phosphorylation at T231 occurs earlier compared to other sites^35,44^, increasing with advancing Braak stage in the transentorhinal region and the medial temporal cortex of AD patients^44,45^. The T231 phospho-residue is present in all types of pathological tau, from pre-fibrillar to the NFT stage^46^. Phosphorylation at S396 and S404, the epitopes recognized by the PHF-1 antibody, has been most commonly associated with fibrillar tau and extracellular NFTs^34,47^. The identification of the aforementioned epitopes in the sarkosyl-insoluble proteome of *APP*_swe_/*PS1*_*dE9*_ mice indicates that murine tau undergoes the complete sequence of phosphorylation events that have been associated with the development of cytoskeletal pathology in AD, substantiating the detection of Gallyas- and thioflavin-S-positive lesions in aged transgenic animals^48^. Although the possibility of phosphorylation at additional tau epitopes should not be excluded, our data highlight pT231, pS238, pS396, pS400 and pS404 as sites of specific interest for targeting pathological tau phosphorylation in familial AD. An imbalance between tau-associated kinases and phosphatases is likely to underlie hyperphosphorylation in the *APP*_swe_/*PS1*_*dE9*_ model. Impaired de-phosphorylation mechanisms appear to be particularly important, as evidenced by the reduced levels of phosphatase 2 A (PP2A) and calcineurin (CaN; PP2B) in 24-month-old transgenic vs. wild-type mice. Both PP2A and CaN are known to interact with PHF tau^49^, de-phosphorylating it at several residues, including pS396 and pS404^50,51^. It is important to note that these enzymes were elevated by physiological ageing in the sarkosyl-insoluble proteome of wild-type animals, which suggests that increasing the level and/or activity of phosphatases may hold potential for the management of tau pathology in AD^52^.

Although the literature on the regulation of tau isoforms in AD remains scarce, a prevalence of 3R tau in the composition of NFTs has been observed in the AD hippocampus by immunohistochemical and biochemical methods^16^. Moreover, a shift from 4R to 3R isoforms has been associated with the morphological evolution of tau-positive neurons from a pre-tangle to the NFT stage^53^. In the present study, a 3R isoform of tau was preferentially sequestered into the sarkosyl-insoluble proteome of *APP*_swe_/*PS1*_*dE9*_ mice, supporting the notion that neurofibrillary pathology is associated with tau isoform imbalances. Similar observations have been made in transgenic mice overexpressing non-mutated 3R *TAU*^54^ or human *TAU* on a *tau* knockout background^8^. These models develop filamentous pathology that is primarily composed of 3R tau, in an age-dependent manner. Thus, any deviation from the physiological ratio of 4R/3R tau is likely sufficient for initiating tau aggregation, in both *TAU* overexpressing and *APP*_swe_/*PS1*_*dE9*_ mice. However, the shift in tau isoform expression occurs downstream of Aβ accumulation in the familial AD model, whereas *TAU* overexpressing mice do not exhibit amyloidosis. In addition, *TAU* in AD is neither overexpressed, nor mutated^55^. Therefore, the pathophysiology that differentiates AD from primary tauopathies is preferentially modeled by *APP*_swe_/*PS1*_*dE9*_ mice, which reproduce the temporal relationship between aberrant APP processing and tau aggregation in familial AD.

The identification of tau isoform-B, which is predominantly expressed in the fetal mouse brain, indicates that immature tau isoforms participate in AD tauopathy^56^, and implicates aberrant transcription and translation mechanisms in the disease process. A re-induction of fetal tau may be attributed to the deregulation of core splicing machinery, which was marked in this study and considered to occur early and selectively in AD^57^. Moreover, as the selection of splice sites is determined by canonical sequences encoded into the genome, the re-expression of fetal isoforms might be a consequence of aberrant cell cycle re-entry^58^. Cell cycle proteins that were deregulated in an age- and genotype-specific manner in this study include Sub1, cdc42, CEND1, Histone H3 and nucleolin (Supplementary Dataset S4). Clearly, the exact mechanisms underlying tauopathy in AD cannot be resolved by the present set of experiments. Our data demonstrate, however, that the formation of PHF tau is associated with loss of regulatory control over *tau* splicing *in vivo*, which may have important implications for the origins and management of tauopathy in AD.

The limitations associated with the overexpression of mutant *APP* and/or *PSEN* have been discussed previously^2^. To exclude the possibility that tauopathy is an artefact of overexpression, it would be important to determine whether it develops in second-generation amyloidosis models, carrying AD-related mutations in physiologically expressed genes. Moreover, it is becoming increasingly recognised that the familial and sporadic forms of AD are not equivalent, despite sharing common clinical and histopathological features^59^. For example, positron emission tomography (PET) with [^11^C]PIB shows accumulation of Aβ fibrils in the cerebellum of familial AD cases, which is not typical of sporadic AD^60^. Cerebellar deposition of hyperphosphorylated tau has been observed in AD cases harbouring the *PSEN1* E280A mutation, but not in sporadic AD^61^. Thus, the pronounced cerebellar involvement in *APP*_*swe*_/*PS1*_*ΔE9*_ mice, which are known to accumulate Aβ in this region^62^, suggests that the model shows face validity for the familial, rather than sporadic forms of AD. Practical considerations in using amyloidosis models to study tau pathology include long waiting times for the accumulation of endogenous murine tau, and mouse-on-mouse/antibody specificity issues, which is a subject of ongoing debate in the tau literature^63,64^. In addition, all the techniques used in this study measured significantly lower levels of tau pathology in *APP*_*swe*_/*PS1*_*ΔE9*_ mice compared to human AD tissue. This was particularly evident in the proteomics data, where levels of sarkosyl-insoluble total tau were not different between *APP*_*swe*_/*PS1*_*ΔE9*_ and control mice, but were > 30-fold elevated in AD vs. non-AD subjects. Studies estimating^65^ and measuring^66^ the amount of insoluble tau protein in the human AD brain show that tau levels are significantly higher than control in symptomatic AD patients, but not in patients with prodromal or preclinical AD. It might thus be that the tau pathology we detect in *APP*_*swe*_/*PS1*_*ΔE9*_ mice corresponds to early stage AD.

Despite these limitations, our data show that Aβ aggregation precedes tau-associated molecular and pathological changes in murine models of familial AD. The progressive accumulation of fibrillar tau can be detected by tools that are employed for the evaluation of PHF tau clinically, such as the Gallyas silver stain and [^18^F]Flortaucipir. The observed pathology occurs in the absence of *TAU* mutations or overexpression, and is characterized by protein hyperphosphorylation and deposition of 3R tau. The aforementioned similarities with human AD argue for repositioning amyloidosis models as tools of translational relevance for the mechanistic study of the interplay between Aβ and tau pathology in AD.

## Methods

### Ethical approval

All methods regarding the use of animal and human tissue were performed in accordance with relevant guidelines and regulations. Mouse tissue: All procedures complied with Danish law (Bekendtgørelse af lov om dyreforsøg, LBK nr 1306 af 23 nov 2007) and European Union directive 2010/63/EU, regulating animal research. Ethical permission was granted by the Animal Ethics Inspectorate of Denmark (nr 2011/561-1950). Human tissue: Fresh-frozen samples from the middle frontal gyrus were obtained from the Maritime Brain Tissue Bank, Department of Medical Neuroscience, Faculty of Medicine, Dalhousie University, Sir Charles Tupper Building, 5850 College Street, Halifax Nova Scotia B3H 4R2. Ethical approval was obtained from the Nova Scotia Health Authority Research Board in Halifax, Canada, and the Danish Biomedical Research Ethical Committee of the region of Southern Denmark (Project ID: S-20070047). Informed, written consent forms were obtained for all subjects.

### Animals and tissue sectioning

*APP*_swe_/*PS1*_ΔE9_ mice express human *APP* harbouring the Swedish double mutations (KM670/671NL) and *PSEN1* lacking exon 9 (*APP*_swe_/*PS1*_ΔE9_), both under control of the mouse prion protein promoter^67^. The mice were originally purchased from the Jackson Laboratories (MMRRC Stock No: 34832-JAX), and were bred and maintained as hemizygotes on a C57BL/6 J background in the Biomedical Laboratory of the University of Southern Denmark. Animals were group-housed (4–8/cage), in a temperature (21 ± 1 °C) and humidity controlled environment (45–65%), under a 12:12 h light:dark cycle (lights on: 7 am). Food and water were available *ad libitum*.

Female *APP*_swe_/*PS1*_ΔE9_ mice were used at 3, 6, 12, and 18 months of age. Sex- and age-matched wild-type (WT) littermates were used as controls. Both male and female mice were used in the 24-month-old groups (n = 6–9/genotype & age-group, total animal number: 71). The animals were euthanized by cervical dislocation, and brains immediately removed and bisected along the midline. Right hemispheres were frozen in isopentane on dry-ice (−30 °C). The olfactory bulb, striatum, cortex, hippocampus, diencephalon, brainstem and cerebellum from the left hemisphere were dissected on a petri dish on ice, collected in Eppendorf^®^ tubes, and frozen on dry-ice. The tissue was stored at −80 °C until use.

Sectioning was carried out at −17 °C using a Leica CM3050S cryostat (Leica Biosystems GmbH). Consecutive, 20 µm-thick sagittal sections were collected at 300 µm intervals. The sections were mounted onto ice-cold Superfrost^TM^ Plus slides (Thermo Fisher Scientific), dried at 4 °C in a box containing silica gel for at least 2 h, and stored at −80 °C for future experiments. Every 13^th^ and 14^th^ section was collected in Eppendorf^®^ tubes for RNA extraction with Trizol^TM^.

Fresh-frozen coronal brain sections of male and female, 20-month-old WT and hemizygote Tg2576 mice, harbouring the Swedish double mutations, were provided by the Centre for Biological Sciences, University of Southampton, U.K. (n = 2–3/group).

### Human tissue

Tissue from the middle frontal gyrus of a sporadic, AD-confirmed patient [BB08-002, Female, 80 years old, *post-mortem* interval (PMI): 9 h, CERAD: Frequent; Braak: V] and a non-AD subject (BB16-023, Female, 83 years old, PMI unknown, CERAD: Sparse; Braak: II) were processed along with the murine samples in order to compare tau pathology between transgenic mouse and human AD tissue. The AD and non-AD samples were chosen for their abundance and complete lack of tau pathology respectively, as assessed by Gallyas silver staining and [^18^F]Flortaucipir autoradiography.

### (Immuno)histochemistry, immunoblotting, ELISA

The Gallyas silver stain was performed according to Kuninaka *et al*.^68^. Thioflavin-S according to Sun *et al*.^48^. Standard protocols were used for tau immunoblotting, immunohistochemistry and ELISA. The following primary antibodies were used: rabbit total Tau (1:1000; A0024, Dako Agilent), rabbit Phospho-Tau Thr231 (1:500; #701056, Thermo Fisher Scientific), rat anti 3R-Tau (1:1000; clone 2A1-1F4; 016-26581, Wako), rabbit Phospho-Ser404 (1:200; OAAF07796, Aviva Systems Biology). Detailed protocols are provided in Supplementary Information.

### Autoradiography and proteomics

[^18^F]Flortaucipir autoradiography was performed according to Marquié *et al*.^69^, proteomics according to Kempf *et al*.^70^ and Thygesen *et al*.^71^. Details are provided in Supplementary Information.

### RT-qPCR

For reverse transcription quantitative polymerase chain reaction (RT-qPCR), Trizol^TM^-isolated RNA (2 μg) from brain sections of WT and TG mice was reverse-transcribed to cDNA, by using the Applied Biosystems^TM^ high-capacity cDNA transcription kit (Thermo Fisher Scientific). Samples were analysed in triplicate on a StepOnePlus^TM^ Real-Time PCR system (Applied Biosystems^TM^, Thermo Fisher Scientific). Each 20 µL sample contained nuclease-free H_2_O (Thermo Fisher Scientific), 1x Maxima SYBR^®^ green/probe master mix (Thermo Fisher Scientific), 500 nM forward and reverse primers (TAG Copenhagen A/S), 4x diluted cDNA for *Mapt*, undiluted cDNA for *Mapt* Isoform-B, and 10x diluted cDNA for hypoxanthine phosphoribosyltransferase (*Hprt1*), which was used as a reference gene. *Hprt1* sequences^72^ and mouse-specific *Mapt* primers spanning exon 10 have been described previously^17^. New primers were designed for isoform-B, forward: CAAGGACAGAGTCCAGTCGAAG; reverse: AAGCAGCTTTTCCCTGCTTGG. Conventional PCR cycling conditions were used [95 °C (10 min), 40 cycles of 95 °C (15 s)/60 °C (1 min)], followed by a melt curve. After normalization to *Hprt1*, data were expressed as fold change from the mean value of the 3-month-old WT samples. Nuclease-free H_2_O and genomic DNA were used as controls.

### Isolation of sarkosyl-insoluble tau

Left brain hemispheres from two mice per group were pooled and weighed (467.1 ± 3.1 mg). The tissue was thoroughly homogenised with a motor driven Potter*-*Elvehjem tissue grinder (WHEATON), in a 5-fold excess (v/w) of 10 mM Tris-HCl buffer (pH 7.4), containing 800 mM NaCl, 1 mM EGTA, 10% sucrose, protease inhibitors (cOmplete^TM^ Protease Inhibitor; Roche Diagnostics) and phosphatase inhibitors (PhosSTOP^TM^; Roche Diagnostics; H buffer). The homogenate was centrifuged at 4 °C for 20 min in a refrigerated ultracentrifuge (27,000 × g; Sorvall RC M150 GX). The supernatant was decanted and kept on ice, the pellet (P1) suspended in 5 vol of H-buffer and re-centrifuged at 27,000 × g for 20 min (4 °C). The combined supernatants (S1) were brought to 1% sarkosyl in H buffer and incubated for 2 h at 37 °C in a C24 incubator shaker (100Rpm; New Brunswick Scientific). Following centrifugation at 200,000 × g for 40 min (4 °C), the sarkosyl-soluble fraction was decanted and kept on ice, and the sarkosyl-insoluble pellet suspended in H buffer, containing 1% CHAPS hydrate (Sigma Aldrich Co.). After filtering through acetate cellulose filters (0.45 µm; VWR International), the filtrates were centrifuged at 200,000 g for 70 min, and the final pellet suspended in 250 µL dH_2_O. Aliquots of 150 µL from the P1, S1, sarkosyl soluble and insoluble fractions were kept for determining tau protein levels. Samples were stored at −80 °C until further processed.

### Tau meso scale

Tau protein concentration in soluble and insoluble fractions was measured with the mouse Total Tau kit (K151DSD-1; Mesoscale Diagnostics LLC). The anti-mouse monoclonal antibody used for detection binds between amino acids 150–200 of Tau, but the clone number and exact epitope recognition site(s) are proprietary. Plates were processed in a SECTOR^®^ Imager 6000 plate reader (Meso Scale Diagnostics LLC), and data acquired with Discovery Workbench software (v.4.0; Meso Scale Diagnostics LLC). Results are presented as pg of tau/mg of total protein, the latter measured at A562 nm with a Pierce^TM^ BCA protein kit and bovine serum albumin as standard (Thermo Fisher Scientific).

### Transmission electron microscopy (TEM) of sarkosyl-insoluble tau

Electron microscopy was performed in the Core Facility for Integrated Microscopy, Faculty of Health and Medical Sciences, University of Copenhagen, Denmark. Carbon-coated copper grids (200 mesh; Ted Pella Inc.) were glow-discharged with a Leica EM ACE 200 (Leica Biosystems Nussloch GmbH), and loaded with 6 µL of sarkosyl-insoluble sample. The sample was adsorbed for 1 min, blotted and stained with 2% phosphotungstic acid in dH_2_0 for 2 min. After blotting and a quick wash with dH_2_0, the samples were examined with a Philips CM 100 TEM (Koninklijke Philips N.V.), operated at an accelerating voltage of 80 kV. Digital images were acquired at a nominal magnification of x180,000, by using an OSIS Veleta digital slow scan 2k × 2k CCD camera and the iTEM software package (Olympus Corporation). Filaments shorter and longer than 200 µm were analysed in at least two fields of view with ImageJ software. Reported values are mean ± SEM of 70 and 32 PHFs for *APP*_swe_/*PS1*_ΔE9_ and AD tissue, respectively.

### Statistical analysis

Parametric testing was employed following inspection of the data for normality with the Kolmogorov-Smirnov test in Prism (v6.01; GraphPad Software). Data sets were analyzed by Statistica^TM^ v10 (TIBCO Software Inc., USA). The effects of age, genotype and brain region on the binding levels of [^18^F]Flortaucipir were analyzed by three-way ANOVA. Gallyas-positive area fraction and tau gene/protein levels were analyzed by two-way ANOVA for the independent factors age and brain region or age and genotype, respectively. Where ANOVA yielded significant effects, Bonferroni *post-hoc* comparisons were used to detect between-group regional and age-dependent differences. Levels of sarkosyl-insoluble tau between 24-month-old TG and WT mice, and PHF dimensions extracted from TG vs. AD brain were compared by two-tailed independent Student’s t-tests. Significance was set at α = 0.05. A 1.3-fold change cut-off value for all TMT ratios was used to rank proteins as up- or down-regulated in the proteomics study^73^.

## Supplementary information

Dataset 1

Supplementary information

## Data Availability

The proteomics data have been deposited to the ProteomeXchange Consortium^74^ via the PRIDE partner repository with the dataset identifier PXD009306 [username: reviewer39090@ebi.ac.uk; password: HdJxxVxU^75^]. The remaining data generated or analysed during this study are included in the article. Raw data are available from the corresponding author upon reasonable request.
