# Supplementary material for: Ageing and amyloidosis underlie the molecular and pathological alterations of tau in a mouse model of familial Alzheimer’s disease
Source: Sci Rep. 2019 Oct 31;9:15758. doi: 10.1038/s41598-019-52357-5 (PMC6823454; doi:10.1038/s41598-019-52357-5)
Supplement: Supplementary file 2 — Supplementary information [file 41598_2019_52357_MOESM2_ESM.pdf]

SUPPLEMENTARY INFORMATION

Ageing and amyloidosis underlie the molecular and pathological alterations of tau in a mouse model of familial Alzheimer's disease

Athanasios Metaxas*, Camilla Thygesen[†], Stefan J. Kempf, Marco Anzalone, Ramanan Vaitheeswaran, Sussanne Petersen, Anne M. Landau, Hélène Audrain, Jessica L. Teeling, Sultan Darvesh, David J. Brooks, Martin R. Larsen, Bente Finsen

[†]Equal contribution

*Corresponding author:

Dr Athanasios Metaxas, Ph.D.

Institute of Molecular Medicine

University of Southern Denmark

Odense DK-5000C

Denmark

Email: ametaxas@health.sdu.dk

SUPPLEMENTARY TABLES

Supplementary Table S1. Evidence of tau hyperphosphorylation in *APP*$_{swe}$/*PS1*$_{\Delta E9}$ mice

Background strain	Age (Months)	Gender	Method of Euthanasia	Method of Tau Evaluation	Antibody/Epitope	Brain Region	Reference
B6.C3	12	Female	Anesthesia	IHC	pS262	NCx	1
B6.C3	~9	Male	Not reported	IF & WB	pS396	NCx & Hip	2
B6.C3	~7.5	Not reported	Anesthesia	IF	AT8 (pS202/pT205)	NCx & Hip	3
B6.C3	6 to >24	Male & Female	Anesthesia	IHC	PHF1, CP13, pT231, p262, pS396, pS422	Entire Brain	4
B6.C3	8	Not reported	Anesthesia	IHC	AT8 (pS202/pT205)	NCx & Hip	5
B6.C3	10	Male	Not reported	WB & Gallyas	AT8 (pS202/pT205)	Entire Brain	6
B6.C3	11 & 18	Not reported	Anesthesia	IHC	AB1518 (not reported)	Hip	7
B6.C3	11	Not reported	Anesthesia	IHC	PHF1 (pS396/pS404)	NCx & Hip	8
B6.C3	~7.5	Not reported	Anesthesia (?)	WB & IHC	AT8 (pS202/pT205) PHF1 (pS369/pS404)	NCx & Hip	9
B6.C3	~7	Male	Anesthesia	WB	pS519, pS202, pS235, pS396, pS404	FrCx & Hip	10
C57BL/6	6	Female	Cervical dislocation	WB	pT205, pS396, pS404	Hip	11
C57BL/6J	22	Female	Anesthesia	WB & IHC	pS199, pS396	NCx & Entire Brain	12
C57BL/6J	7	Male	Not reported	IF	pS199	NCx & Hip	13
C57BL/6J	3-12	Not reported	Not reported	WB	PHF1 (pS396/pS404)	FrCx	14
C57BL/6J	12	Male	Cervical dislocation	Proteomics & Gallyas	N/A	NCx & Hip, Olf. Bulb, Brainstem	15
C57BL/6	~9	Female	Anesthesia	WB	pS235, pT205	Entire Brain	16
C57BL/6J	~7.5	Male	Not reported	WB	pS262	NCx & Hip	17
C57BL/6	7	Male	Decapitation	WB	PHF1 (pS396/pS404)	Hip	18
C57BL/6	~12	Female	Anesthesia	WB	pS235, pT205	NCx & Hip	19
C57BL/6	3 & 6	Male	Not reported	WB	pS199, pT205, pS396, pS404	Hip	20
C57BL/6J	~7.5	Male	Not reported	IF	pT181	NCx & Hip	21
C57BL/6J	6 & 9	Male	Anesthesia	IF	pS199, pS202, pS262, pT181	NCx	22
C57BL/6	6-7	Not reported	Anesthesia	IHC	AT8 (pS202/pT205)	Amygdala	23
C57BL/6J	12	Not reported	Anesthesia	IHC/IF	AT8 (pS202/pT205) PHF1 (pS369/pS404)	NCx	24
Not reported	~12	Male	Anesthesia	WB	pS235, pT205	Entire Brain	25
Not reported	3-12	Not reported	Anesthesia	WB	pS199, pT205, pS396, pS404	Entire Brain	26
Not reported	6	Not reported	Anesthesia	WB	AT8 (pS202/pT205)	NCx	27
Not reported	7	Not reported	Anesthesia	WB	pS396	Brain hemisphere	28
Not reported	~7	Not reported	Anesthesia	WB	AT8 (pS202/pT205)	NCx	29
Not reported	>18	Not reported	Anesthesia	IHC	pS199, pT231	Cerebellum	30
Not reported	~2-3	Male	Anesthesia	WB	PHF1 (pS396/pS404)	NCx & Hip	31
Not reported	2	Male	Anesthesia	WB	AT8 (pS202/pT205) PHF1 (pS396/pS404)	Subventricular zone, NCx & Hip	32

Abbreviations: IHC: Immunohistochemistry; WB: Western Blot; IF: Immunofluorescence; NCx: Neocortex; Fr Cx: Frontal Cortex; Hip: Hippocampus.

References to Supplementary Table S1

1. Metaxas, A., *et al.* Reduced Serotonin Transporter Levels and Inflammation in the Midbrain Raphe of 12 Month Old APPswe/PSEN1dE9 Mice. *Current Alzheimer research* **15**, 420-428 (2018).

2. Li, C., *et al.* Thamnolia vermicularis extract improves learning ability in APP/PS1 transgenic mice by ameliorating both Abeta and Tau pathologies. *Acta Pharmacol Sin* **38**, 9-28 (2017).

3. Tapia-Rojas, C., Aranguiz, F., Varela-Nallar, L. & Inestrosa, N.C. Voluntary Running Attenuates Memory Loss, Decreases Neuropathological Changes and Induces Neurogenesis in a Mouse Model of Alzheimer's Disease. *Brain Pathol* **26**, 62-74 (2016).

4. Li, T., *et al.* The neuritic plaque facilitates pathological conversion of tau in an Alzheimer's disease mouse model. *Nature communications* **7**, 12082 (2016).

5. Huang, X.T., *et al.* Reducing iron in the brain: a novel pharmacologic mechanism of huperzine A in the treatment of Alzheimer's disease. *Neurobiology of aging* **35**, 1045-1054 (2014).

6. Barbero-Camps, E., Fernandez, A., Martinez, L., Fernandez-Checa, J.C. & Colell, A. APP/PS1 mice overexpressing SREBP-2 exhibit combined Abeta accumulation and tau pathology underlying Alzheimer's disease. *Hum Mol Genet* **22**, 3460-3476 (2013).

7. Carrera, I., *et al.* Vaccine Development to Treat Alzheimer's Disease Neuropathology in APP/PS1 Transgenic Mice. *Int J Alzheimers Dis* **2012**, 376138 (2012).

8. Cancino, G.I., *et al.* c-Abl tyrosine kinase modulates tau pathology and Cdk5 phosphorylation in AD transgenic mice. *Neurobiology of aging* **32**, 1249-1261 (2011).

9. Inestrosa, N.C., *et al.* Tetrahydrohyperforin prevents cognitive deficit, Abeta deposition, tau phosphorylation and synaptotoxicity in the APPswe/PSEN1DeltaE9 model of Alzheimer's disease: a possible effect on APP processing. *Transl Psychiatry* **1**, e20 (2011).

10. Ding, Y., *et al.* Retinoic acid attenuates beta-amyloid deposition and rescues memory deficits in an Alzheimer's disease transgenic mouse model. *The Journal of neuroscience : the official journal of the Society for Neuroscience* **28**, 11622-11634 (2008).

11. Ettcheto, M., *et al.* Dexibuprofen prevents neurodegeneration and cognitive decline in APPswe/PS1dE9 through multiple signaling pathways. *Redox Biol* **13**, 345-352 (2017).

12. Bu, X.L., *et al.* Blood-derived amyloid-beta protein induces Alzheimer's disease pathologies. *Molecular psychiatry* (2017).

13. Yang, S.H., *et al.* Nec-1 alleviates cognitive impairment with reduction of Abeta and tau abnormalities in APP/PS1 mice. *EMBO Mol Med* **9**, 61-77 (2017).

14. Zhang, J., *et al.* Tissue Transglutaminase and Its Product Isopeptide Are Increased in Alzheimer's Disease and APPswe/PS1dE9 Double Transgenic Mice Brains. *Mol Neurobiol* **53**, 5066-5078 (2016).

15. Kempf, S.J., *et al.* An integrated proteomics approach shows synaptic plasticity changes in an APP/PS1 Alzheimer's mouse model. *Oncotarget* **7**, 33627-33648 (2016).

16. Du, Y., *et al.* Morin reverses neuropathological and cognitive impairments in APPswe/PS1dE9 mice by targeting multiple pathogenic mechanisms. *Neuropharmacology* **108**, 1-13 (2016).

17. Jeon, S., *et al.* Illite improves memory impairment and reduces Abeta level in the Tg-APPswe/PS1dE9 mouse model of Alzheimers disease through Akt/CREB and GSK-3beta phosphorylation in the brain. *J Ethnopharmacol* **160**, 69-77 (2015).

18. Vargas, J.Y., Ahumada, J., Arrazola, M.S., Fuenzalida, M. & Inestrosa, N.C. WASP-1, a canonical Wnt signaling potentiator, rescues hippocampal synaptic impairments induced by Abeta oligomers. *Experimental neurology* **264**, 14-25 (2015).

19. Zhou, Q., *et al.* Inhibition of c-Jun N-terminal kinase activation reverses Alzheimer disease phenotypes in APPswe/PS1dE9 mice. *Annals of neurology* **77**, 637-654 (2015).

20. Pedros, I., *et al.* Early alterations in energy metabolism in the hippocampus of APPswe/PS1dE9 mouse model of Alzheimer's disease. *Biochim Biophys Acta* **1842**, 1556-1566 (2014).

21. Aso, E., Juves, S., Maldonado, R. & Ferrer, I. CB2 cannabinoid receptor agonist ameliorates Alzheimer-like phenotype in AbetaPP/PS1 mice. *Journal of Alzheimer's disease : JAD* **35**, 847-858 (2013).

22. Aso, E., *et al.* Amyloid generation and dysfunctional immunoproteasome activation with disease progression in animal model of familial Alzheimer's disease. *Brain Pathol* **22**, 636-653 (2012).

23. Li, L., Cheung, T., Chen, J. & Herrup, K. A comparative study of five mouse models of Alzheimer's disease: cell cycle events reveal new insights into neurons at risk for death. *Int J Alzheimers Dis* **2011**, 171464 (2011).

24. Liu, Y., *et al.* Amyloid pathology is associated with progressive monoaminergic neurodegeneration in a transgenic mouse model of Alzheimer's disease. *The Journal of neuroscience : the official journal of the Society for Neuroscience* **28**, 13805-13814 (2008).

25. Wei, C., *et al.* Mithramycin A Alleviates Cognitive Deficits and Reduces Neuropathology in a Transgenic Mouse Model of Alzheimer's Disease. *Neurochem Res* **41**, 1924-1938 (2016).

26. Porquet, D., *et al.* Amyloid and tau pathology of familial Alzheimer's disease APP/PS1 mouse model in a senescence phenotype background (SAMP8). *Age (Dordr)* **37**, 9747 (2015).

27. Ramos-Rodriguez, J.J., *et al.* Prediabetes-induced vascular alterations exacerbate central pathology in APPswe/PS1dE9 mice. *Psychoneuroendocrinology* **48**, 123-135 (2014).

28. Shi, J.Q., *et al.* Antiepileptics topiramate and levetiracetam alleviate behavioral deficits and reduce neuropathology in APPswe/PS1dE9 transgenic mice. *CNS Neurosci Ther* **19**, 871-881 (2013).

29. Ramos-Rodriguez, J.J., Molina-Gil, S., Rey-Brea, R., Berrocoso, E. & Garcia-Alloza, M. Specific serotonergic denervation affects tau pathology and cognition without altering senile plaques deposition in APP/PS1 mice. *PloS one* **8**, e79947 (2013).

30. Lomoio, S., *et al.* Cerebellar amyloid-beta plaques: disturbed cortical circuitry in AbetaPP/PS1 transgenic mice as a model of familial Alzheimer's disease. *Journal of Alzheimer's disease : JAD* **31**, 285-300 (2012).

31. Hu, Y.S., *et al.* Complex environment experience rescues impaired neurogenesis, enhances synaptic plasticity, and attenuates neuropathology in familial Alzheimer's disease-linked APPswe/PS1DeltaE9 mice. *FASEB J* **24**, 1667-1681 (2010).

32. Demars, M., Hu, Y.S., Gadadhar, A. & Lazarov, O. Impaired neurogenesis is an early event in the etiology of familial Alzheimer's disease in transgenic mice. *J Neurosci Res* **88**, 2103-2117 (2010).

SUPPLEMENTARY FIGURES

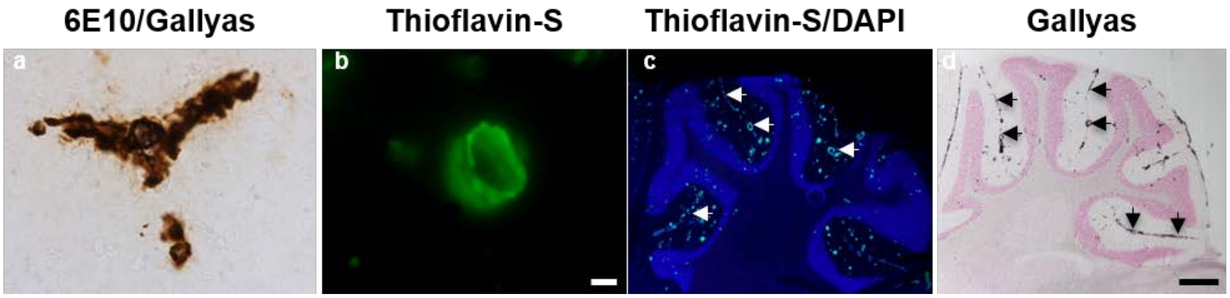

Supplementary Fig. S1. Vascular pathology in *APP*$_{swe}$/*PS1*$_{\Delta E9}$ mice

Vascular and meningeal lesions in 18-month-old *APP*$_{swe}$/*PS1*$_{\Delta E9}$ mice. Gallyas/6E10- (a) and thioflavin-S-positive vascular pathology (b). The arrows in (c) & (d) respectively point to thioflavin-S and Gallyas signal in the meninges of the cerebellum. Scale bars: 10 µm (a & b), 200 µm (c & d).

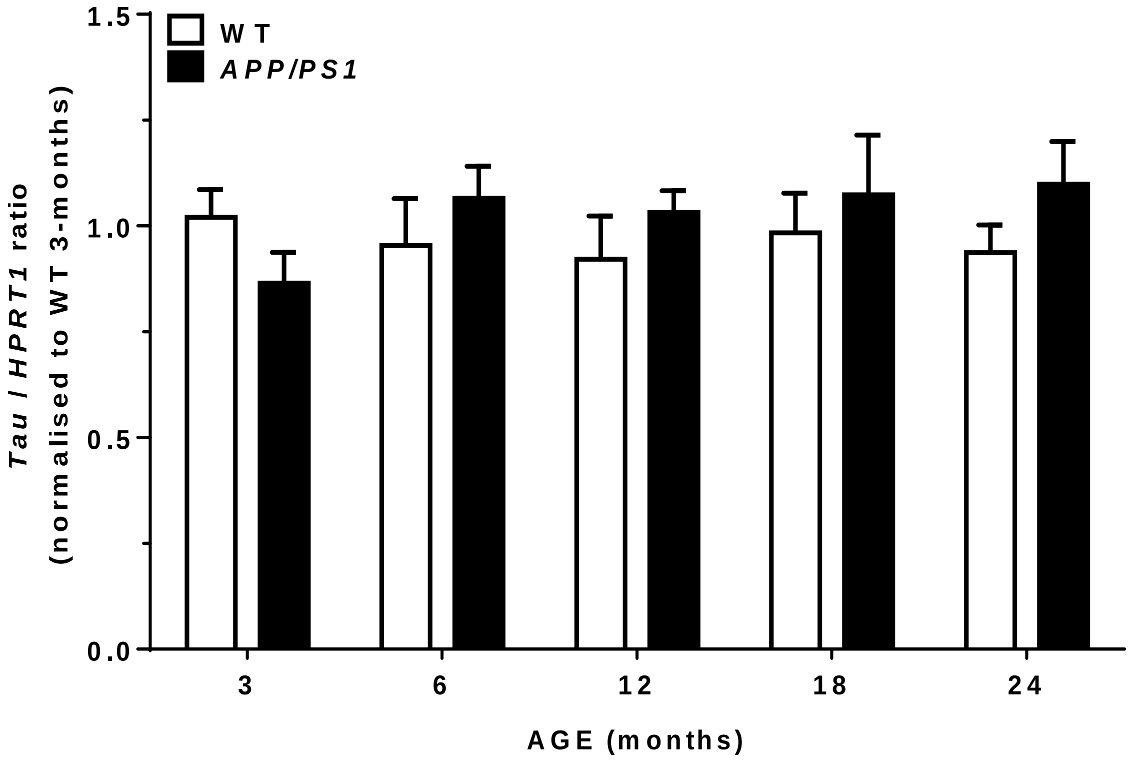

Supplementary Fig. S2. Regulation of *Mapt* mRNA in wild-type (WT) and transgenic mice

Levels of endogenous murine *tau* mRNA were not altered by age [$F_{(4,50)}$=0.29, *P*>0.05] or genotype [$F_{(1,50)}$=0.93, *P*>0.05]. PCR products of x4 diluted cDNA were determined after 24 cycles. A single peak was obtained by melt-curve analysis, and no signal detected in the genomic DNA and buffer controls. The efficiency of amplification was 99.2±0.2% for *Hprt1* and 100.3±2.1% for *Mapt*.

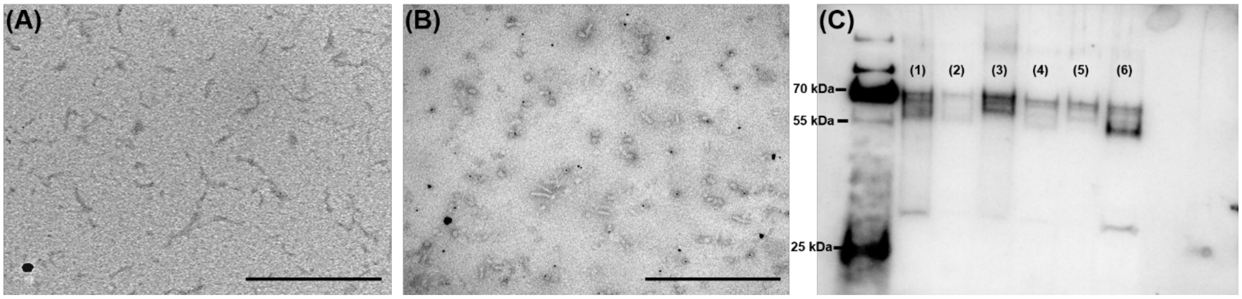

Supplementary Fig. S3. TEM and immunoblotting of sarkosyl-insoluble tau. Filaments were extracted according to **(A)** Sahara et al. [1] and **(B)** Greenberg and Davies [2]. Scale bar: 500 nm. **(C)** A triplet of immunoreactive bands near the 55-70 kDa range was detected by both methods, by using a rabbit antibody directed to the C-terminal domain of unmodified tau (aa 243-441; A0024, Dako Agilent). Total tau immunoreactivity is shown for the following groups: (1) TG 24 months, (6) WT 24 months (Greenberg and Davies method). (2) WT 24 months, (3) Human AD, (4) TG 18 months, (5) TG 24 months (Sarhara et al. method). Note that under the Greenberg and Davies method, a relatively more intense band of lower molecular weight (55 kDa) was observed in 24-month-old WT mice (6), as compared to the more intense bands of higher molecular weight (70 kDa) in 24-month-old TG mice (1).

A

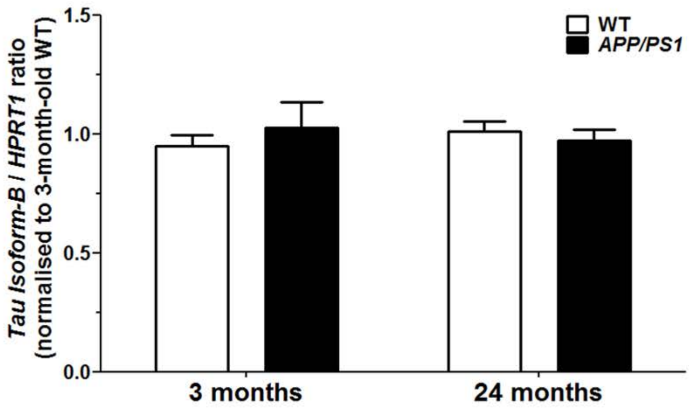

B

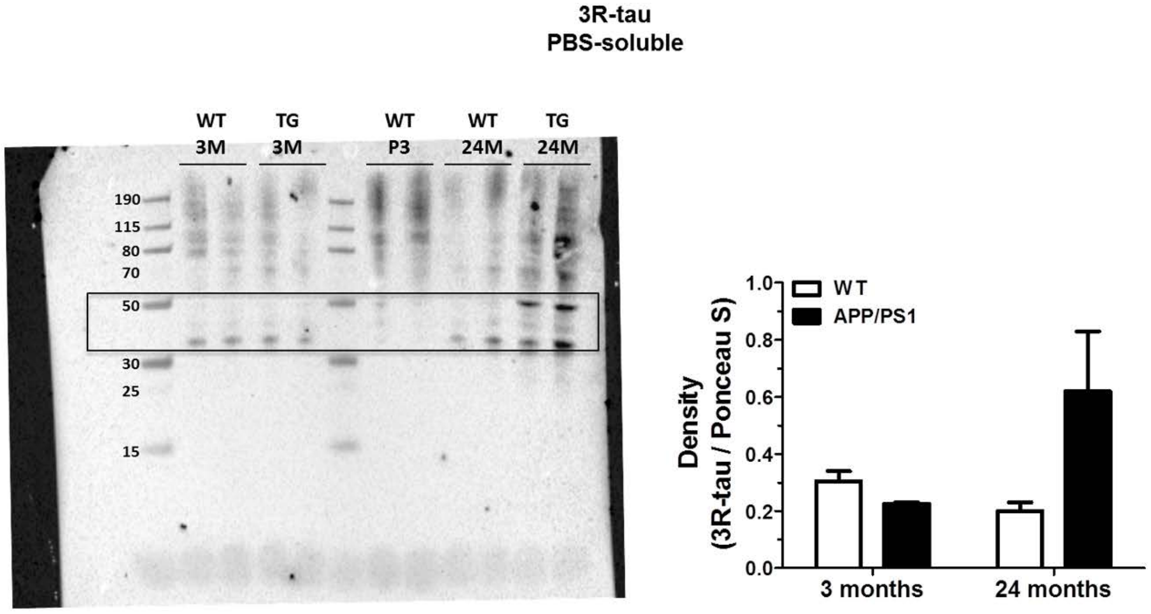

Supplementary Fig. S4. RT-qPCR and immublotting of tau isoform-B. (A) Regulation of isoform-B *Mapt* mRNA in wild-type (WT) and transgenic (TG) $APP_{swe}/PS1_{\Delta E9}$ mice. The expression of minor fetal *tau* was not altered by age [$F_{(1,20)}=0.01$, $P>0.05$] or genotype [$F_{(1,20)}=0.09$, $P>0.05$; Two-way ANOVA]. PCR products of undiluted cDNA were determined after 30 cycles. A single peak was obtained by melt-curve analysis, and no signal was detected in the genomic DNA and buffer controls. The efficiency of amplification was 92% for *Hprt1* and

110% for *Mapt* isoform-B. **(B)** Western blot of PBS-soluble 3R tau in the neocortex of 3 and 24-month-old WT and TG mice, and in postnatal day 3 C57Bl/6J mouse brain (P3). Three bands in the 35-50 kDa range were detected in all groups, and were most intensely labelled in 24-month-old TG mice. High molecular weight smears were present in all groups, particularly in neonatal mice. Quantification of 3R tau lanes was based on total protein content, which was calculated by Ponceau S staining. Results are expressed as mean ± SEM of n=2 animals/group. There were no effects of age [$F_{(1,4)}=1.8$, $P>0.05$] and genotype [$F_{(1,4)}=2.5$, $P>0.05$] on the levels of 3R tau by two-way ANOVA.

References to Supplementary Figures

1. Sahara, N., *et al.* Assembly of tau in transgenic animals expressing P301L tau: alteration of phosphorylation and solubility. *Journal of neurochemistry* **83**, 1498-1508 (2002).

2. Greenberg, S.G. & Davies, P. A preparation of Alzheimer paired helical filaments that displays distinct tau proteins by polyacrylamide gel electrophoresis. *Proceedings of the National Academy of Sciences of the United States of America* **87**, 5827-5831 (1990).

SUPPLEMENTARY METHODS

Gallyas silver staining

The Kuninaka et al. modification of the Gallyas silver method was used to examine neurofibrillary pathology [1,2]. Fresh-frozen sections were directly immersed in 4% neutral buffered formalin (NBF) at 4°C for 24 h. The sections were thoroughly washed in ultrapure deionized H_2O (dH_2O; Ultra Clear™, Siemens), dried at room temperature (RT) for 10 min and defatted for 1 h in a solution of chloroform/99% ethanol (1:1) in the dark. Following hydration through a series of graded ethanols into dH_2O (2 x 1 min: 99%, 96%, 70%), slides were immersed into an aqueous solution of 0.25% potassium permanganate (20 min), washed in dH_2O (1 min), and incubated in 1% oxalic acid (2 min). After washing in dH_2O (2 x 5 min), sections were transferred into the alkaline silver iodide solution (1 min), washed with 0.5% acetic acid (2 x 5 min), and developed in a water bath at 15°C, until the appearance of a brownish shade (12-14 min). The developed sections were washed in 0.5% acetic acid (3 min), toned with 0.1% gold chloride (10 min), fixed with 1% sodium thiosulfate (5 min), and counterstained with 0.1% nuclear fast red (3 min). Ethanol and chloroform were from VWR International. The remaining chemicals were from Sigma-Aldrich Co.

Aβ immunohistochemistry on silver-stained sections

The biotinylated 6E10 antibody (SIG-39340, Nordic BioSite) was used to investigate the association between neurofibrillary pathology and Aβ load in transgenic mouse and human AD tissue. Clone 6E10 is raised against amino acids 1-16 of human Aβ, recognizing multiple amyloid peptides and precursor forms (manufacturer information). Silver-stained sections were immersed in 70% formic acid for 30 min, rinsed for 10 min in 50 mM Tris-buffered saline (TBS, pH 7.4), and further washed/permeabilised in TBS containing 1% Triton X-100 (3 x 15 min). Sections were subsequently blocked for 30 min in

TBS, containing 10% fetal bovine serum (FBS). Incubation with the 6E10 anti-β-amyloid antibody was carried out overnight at 4°C, in TBS containing 10% FBS (1:500 dilution of stock). Adjacent, negative control sections were incubated with biotin-labelled mouse IgG1 (MG115, Thermo Fisher Scientific), diluted to the same protein concentration as the primary antibody. Following incubation with 6E10, the sections were adjusted to RT for 30 min and washed in TBS+1% Triton X-100 (3 x 15 min). Endogenous peroxidase activity was quenched for 20 min in a solution of TBS/methanol/H_2O_2 (8:1:1). After washing in TBS+1% Triton X-100 (3 x 15 min), sections were incubated for 3 h with HRP-streptavidin in TBS/10% FBS (1:200; GE Healthcare Life Sciences). After a final wash in TBS (3 x 10 min), peroxidase activity was visualised with 0.05% 3,3′diaminobenzidine (DAB) in TBS buffer, containing 0.01% H_2O_2 (Sigma Aldrich Co.).

For all light microscopy studies, the developed sections were thoroughly washed in dH_2O, dehydrated in graded alcohols, cleared in xylene, and cover-slipped with PERTEX® (Histolab Products AB).

Thioflavin-S staining

Thioflavin-S staining was performed according to Sun et al. [3]. Fresh-frozen sections were directly immersed in 4% NBF at 4°C for 24 h. The sections were thoroughly washed in ultrapure dH_2O, dried at RT for 10 min, and defatted for 1 h in a solution of chloroform/99% ethanol (1:1) in the dark. Following hydration through a series of graded ethanols into dH_2O (2 x 1 min: 99%, 96%, 70%), slides were immersed into an aqueous solution of 0.25% potassium permanganate (5 min), washed in dH_2O (1 min), and incubated in 1% oxalic acid (2 min). After washing in dH_2O (2 x 2.5 min), the sections were incubated with freshly-prepared 0.25% $NaBH_4$ (2 x 5 min), washed in dH_2O (5 x 2 min), and transferred into a 0.1% thioflavin-S solution (8 min; dark incubation). The sections were differentiated

in 80% ethanol (2 x 10 s), washed in dH_2O (3 x 5 dips), and incubated for 30 min at 4ºC in the dark with high-concentration phosphate buffer, to prevent photobleaching (411 mM NaCl, 8.1 mM KCl, 30 mM Na_2HPO_4; 5.2 mM KH_2PO_4). Following a dip in dH_2O, the sections were counterstained with 30 µM DAPI (4',6-diamidino-2-phenylindole) for 10 min and mounted with Aquatex® mounting medium (Sigma Aldrich Co.).

Photomicrographs were acquired with an Olympus DP71 digital camera, mounted on an Olympus BX51 microscope equipped for Epi-Fluorescence illumination, or an Olympus DP80 Dual Monochrome CCD camera, mounted on a motorized BX63 Olympus microscope.

Immunoblotting of sarkosyl-insoluble tau

Ten µg of lysed, denatured protein were separated on 4-12% Bolt Bis-Tris gradient gels (NW04125Box, Novex®), and transferred to polyvinylidene fluoride (PVDF) membranes using the Trans-Blot SD Semi-Dry Transfer Cell system (Bio-Rad Laboratories Inc.). Protein content on the PVDF membranes was visualized with Ponceau S. Following washing (10 min) and blocking for 1 h in either Roti®-Block (Carl Roth GmbH) or 5% skimmed milk, the membranes were incubated overnight at 4°C in blocking solution, containing rabbit primary antibodies for unmodified tau (1:1000; A0024, Dako Agilent) and tau phopshorylated at serine 404 (pS404; 1:200; OAAF07796, Aviva Systems Biology), or rat anti-3R tau antibody (1:1000; 016-26581, Wako). The blots were washed in TBS+1% Triton X-100 (3 x 15 min; TBS-Tx) and incubated for 2 h with horseradish peroxidase (HRP)-conjugated secondary antibody (anti-rabbit IgG, HRP-linked antibody, #7074; Cell Signaling Technology® or anti-rat peroxidase antibody, A9037, Sigma Aldrich Co.; 1:5000). After a final wash in TBS-Tx (3 x 15 min), the blots were developed with enhanced chemiluminescent substrate (ECL),

according to manufacturer instructions (Luminata™ Forte Western HRP Substrate, WBLUF0100, Merck Millipore).

Immunohistochemistry of pT231 tau

Free-floating, 50 µm-thick, coronal vibratome sections from the brain of 3- and 18-month-old *APP*$_{swe}$/*PS1*$_{\Delta E9}$ and WT animals (n=3-4/group) were available for these experiments [10]. The sections were washed in TBS overnight at 4ºC to remove the de Olmos cryoprotectant. Endogenous peroxidase activity was quenched in TBS/methanol/30% H_2O_2 (8:1:1) for 30 min at RT, and the sections washed in TBS, containing 1% Triton X-100 (TBS-Tx; 4 x 30 min). After blocking in TBS-Tx containing 10% FBS for 1 h, the sections were incubated with rabbit pT231 antibody (1:500; #701056, Thermo Fisher Scientific), at RT for 1 h and overnight at 4ºC. After washing in TBS-Tx, the sections were incubated for 2 h at RT using the EnVision+ System-HRP Labelled Polymer (K4003; Dako Agilent). Following washes in TBS, peroxidase activity was detected with 0.05% DAB in TBS buffer (pH 7.4), containing 0.01% H_2O_2 (Sigma Aldrich Co.). The developed sections were washed thoroughly in dH_2O, dried on glass slides, dehydrated in graded alcohols, cleared in xylene, and cover-slipped with PERTEX® (Histolab Products AB).

pT231 and 3R tau ELISA

New samples were generated for these studies, using brain tissue from 3- and 24-month-old, *APP*$_{swe}$/*PS1*$_{\Delta E9}$ TG and WT control mice (n=2-3/group). The isolation of sarkosyl-insoluble tau from the left-brain hemisphere was performed as described in section ‘Isolation of sarkosyl-insoluble tau’. Soluble tau was isolated from the neocortex of the right brain hemisphere, by homogenizing tissue in ice-cold sterile PBS, supplemented with protease and phosphatase inhibitors (Roche Diagnostics),

using an ULTRA-TURRAX T25 basic homogenizer (Ika Werke). The homogenates were centrifuged at 9000 x *g* for 20 min at 4 °C, and the supernatants collected and stored at −80 °C until use.

Experimental samples were diluted as needed in 15 mM sodium carbonate/35 mM sodium bicarbonate buffer (pH 9.6). High-bind, 96 micro-well plates (nr: 82.1581.200, Hounisen) were coated with 100 µL sample and incubated overnight at 4°C under mild shaking. The plates were washed four times in PBS containing 0.05% Tween-20 (PBS-T), and blocked with Blocker™ Casein in PBS (nr: 37582, Thermo Fisher Scientific) for 1 h at RT. After washing in PBS-T, rabbit anti-mouse phospho-threonine 231 (pT231; #701056, Thermo Fisher Scientific) and rat anti-3R tau (016-26581, Wako) were diluted 1:1000 in PBS containing 10% FBS, and added to the plates at RT for 1 h. Rabbit Ig (X0903, Dako Agilent) and rat $IgG2_b$ (#02-9288, Thermo Fisher Scientific), diluted to the same protein concentration as the primary antibodies, were used for control. Following washes in PBS-T, the plates were incubated for 1 h at RT with biotinylated donkey anti-rabbit IgG for pT231 (RPN1004, GE Healthcare Life Sciences) or biotinylated goat anti-rat IgG for 3R tau (#31830, Thermo Fisher Scientific), which were diluted 1:500 in PBS containing 10% FBS. Plates were subsequently washed with PBS-T, and incubated for 30 min with streptavidin-HRP conjugate (1:500; RPN 1231, GE Healthcare Life Sciences). After washing, plates were developed in the dark with the Pierce™ TMB Substrate Kit (nr 34021, Thermo Fisher Scientific). The reactions were stopped with 2N H_2SO_4 and the signal read by a Tecan Sunrise™ microplate reader at 450 nm (570 nm for reference signal).

For pT231, standard curves were constructed by two-fold serial dilutions of pooled sample from the neocortex of 24-month-old $APP_{swe}/PS1_{\Delta E9}$ mice. For 3R tau, brains from postnatal day 3 C57BL/6J

mice were used to construct the standard curves. Results are expressed as arbitrary units (U), normalized to total protein concentration (mg/mL), the latter determined with a BCA protein kit.

[^{18}F]Flortaucipir autoradiography

Autoradiography experiments were conducted at the Department of Nuclear Medicine and PET-centre, Aarhus University, Denmark. [^{18}F]Flortaucipir was synthesised in Aarhus using previously detailed methods [4].

[^{18}F]Flortaucipir autoradiography was performed as described previously [5], with minor modifications. Sections were thawed to RT for 20 min and fixed/permeabilised in 100% methanol for 20 min. The sections were incubated for a period of 60 min in a 160 mL bath of 10 mM phosphate buffered saline (PBS, pH 7.4), containing 38.4±9.6 MBq [^{18}F]Flortaucipir (specific activity: 145±68 GBq/µmol). A series of adjacent brain sections was incubated with identical amounts of radioligand in the presence of 50 µM 'cold' flortaucipir, to assess non-specific binding (NSB). Following incubation, sections were serially washed in PBS (1 min), 70% ethanol in PBS (2 x 1min), 30% ethanol in PBS (1 min) and PBS (1 min). After rapid drying under a stream of cold air, the sections were placed in light-tight cassettes and exposed against FUJI multi-sensitive phosphor screens for 30 min (BAS-IP SR2025, GE Healthcare Life Sciences). To allow quantification, standards of known radioactive concentration were prepared by serial dilution of the [^{18}F]Flortaucipir incubation solution, and exposed along with the sections. Images were developed in a BAS-5000 phosphor-imager at 25 µm resolution.

For image analysis, the intensity values produced by the ^{18}F standards were entered with their corresponding radioactivity values (kBq/mL) into a calibration table, and the relationship between

radioactivity and intensity determined by using ImageJ software (v. 1.51c; National Institutes of Health, USA). Adjustments were undertaken to allow for the radioactive decay of [^{18}F]Flortaucipir. Values of specific binding were derived after subtraction of NSB from total binding images.

Mass spectrometry-based proteomics of sarkosyl-insoluble tau

Workflows for protein reduction, alkylation, and enzymatic digestion have been described in detail [6]. Labelling of tryptic peptides with tandem mass tag (TMT) reagents was performed as per manufacturer instructions (AB Sciex Pte. Ltd.): TMT-126 for WT-3 months(1), TMT-127N WT-3 months(2), TMT-127C TG-3 months(1), TMT-128N TG-3 months(2), TMT-128C for WT-24 months(1), TMT-129N for WT-24 months(2), TMT-129C for TG-24 months(1), 130N for TG-24 months(2), TMT-130C for human non-AD and TMT-131 for human AD. Human AD and non-AD samples were included for validation, as well as for taking advantage of the stacking effect of the TMT10-plex, in order to increase identification rates. The labelled peptides from all groups were mixed 1:1, dried down and stored for further enrichment and analysis.

Equal volumes of the labelled peptides were pooled into a master mix and subjected to a modified TiO_2 workflow[7], followed by sample desalting, fractionation[8] by hydrophilic interaction liquid chromatography (HILIC), and tandem mass spectrometry with a Q Exactive™ HF Hybrid Quadrupole-Orbitrap™ mass spectrometer (Thermo Fisher Scientific). Briefly, TMT-labelled mixtures were suspended into 0.1% FA and separated on an analytical ReproSil-Pur C18 AQ column (Dr. Maisch GmbH), packed in-house (17 cm x 75 µm; 3 µm) and operated on an EASY-nanoLC system (Thermo Fisher Scientific), at a flow rate of 250 nL/min. The eluent was directed toward the ion transfer tube of the Orbitrap instrument by dynamic electrospray ionization. The Orbitrap acquired the full MS scan

with an automatic gain control target value of $3x10^6$ ions and a maximum fill time of 100 ms. Each MS scan was acquired at high-resolution [120,000 full-width half maximum (FWHM)] at m/z 200, with a mass range of 400-1400 Da. The 12 most abundant peptide ions were selected for higher energy collision-induced dissociation fragmentation (collision energy: 34 V) if they were at least doubly-charged. Fragmentation was performed at high resolution (60,000 FWHM) for a target of $1x10^5$ and a maximum injection time of 60 ms using an isolation window of 1.2 m/z and a dynamic exclusion of 20s.

Raw data were searched against the mus musculus or homo sapiens reference databases from swissprot and uniprot via Mascot (v2.3.02, Matrix Science) and Sequest HT search engines, respectively, using Proteome Discoverer (v2.1, Thermo Fisher Scientific). A precursor mass tolerance of 20 ppm and a product ion mass tolerance of 0.05 Da was applied, allowing two missed cleavages for trypsin. Fixed modifications included carbamidomethylation of Cys/Arg and TMT-10plex labeling for Lys and N-termini. Variable modifications contained phosphorylation on Ser/Thr/Tyr, acetylation on the N-termini, oxidation of Met and deamidation of Asn. The TMT datasets were quantified using the centroid peak intensity with the ‘reporter ions quantifier’ node. To ensure high-confident identification, we used the Mascot percolator algorithm (q value filter set to 0.01), Mascot and Sequest HT peptide rank 1, a cut-off Mascot score value of ≥18, and a Sequest HTΔCn value of 0.1. Only high confident peptides were used for further analysis. Subsequently, the peptides were filtered against a Decoy database resulting into a false discovery rate (FDR) of < 0.01. Two pooled murine biological replicates per group without missing values were considered for the analysis, and normalization was performed on the protein median. Tau isoform-specific searches were performed by creating a Uniprot database of isoform sequences for mouse Tau (Uniprot ID: P10637-1, -2, -3. -4, -5, -6) and human Tau (Uniprot ID:

P10636-1, -2, -3, -4, -5, -6, -7, -8, -9), as performed by Morris et al. [9]. Moderate confidence peptides (FDR<0.05) were included in the isoform-specific search.

References to Supplementary Methods

1. Gallyas, F. Silver staining of Alzheimer's neurofibrillary changes by means of physical development. *Acta Morphol Acad Sci Hung* **19**, 1-8 (1971).

2. Kuninaka, N., *et al.* Simplification of the modified Gallyas method. *Neuropathology* **35**, 10-15 (2015).

3. Sun, A., Nguyen, X.V. & Bing, G. Comparative analysis of an improved thioflavin-s stain, Gallyas silver stain, and immunohistochemistry for neurofibrillary tangle demonstration on the same sections. *J Histochem Cytochem* **50**, 463-472 (2002).

4. Shoup, T.M., *et al.* A concise radiosynthesis of the tau radiopharmaceutical, [(18) F]T807. *J Labelled Comp Radiopharm* **56**, 736-740 (2013).

5. Marquie, M., *et al.* Validating novel tau positron emission tomography tracer [F-18]-AV-1451 (T807) on postmortem brain tissue. *Annals of neurology* **78**, 787-800 (2015).

6. Kempf, S.J., *et al.* An integrated proteomics approach shows synaptic plasticity changes in an APP/PS1 Alzheimer's mouse model. *Oncotarget* **7**, 33627-33648 (2016).

7. Engholm-Keller, K., *et al.* TiSH--a robust and sensitive global phosphoproteomics strategy employing a combination of TiO2, SIMAC, and HILIC. *J Proteomics* **75**, 5749-5761 (2012).

8. Melo-Braga, M.N., Ibanez-Vea, M., Larsen, M.R. & Kulej, K. Comprehensive protocol to simultaneously study protein phosphorylation, acetylation, and N-linked sialylated glycosylation. *Methods Mol Biol* **1295**, 275-292 (2015).

9. Morris, M., *et al.* Tau post-translational modifications in wild-type and human amyloid precursor protein transgenic mice. *Nat Neurosci* **18**, 1183-1189 (2015).

10. Severino, M., *et al.* Established amyloid-beta pathology is unaffected by chronic treatment with the selective serotonin reuptake inhibitor paroxetine. *Alzheimers Dement (N Y)* **4**, 215-223 (2018).
